# CD317^+^ MSCs expanded with chemically defined media have enhanced immunological anti-inflammatory activities

**DOI:** 10.1186/s13287-023-03618-8

**Published:** 2024-01-02

**Authors:** Jun Song, Qi Ma, Yumeng Li, Xianqi Wang, Si Chen, Bowei Liang, Xiaoqi Lin, Jieting Chen, Shiru Xu, Shaoquan Shi, Jingting Zhang, Lianghui Diao, Yong Zeng, Jianyong Xu

**Affiliations:** 1 Key Laboratory of Animal Cellular and Genetic Engineering of Heilongjiang Province, College of Life Science, Northeast Agricultural University, Harbin, 150000 People’s Republic of China; 2 Shenzhen Key Laboratory of Reproductive Immunology for Peri-Implantation, Shenzhen Zhongshan Institute for Reproduction and Genetics, Shenzhen Zhongshan Obstetrics and Gynecology Hospital (Formerly Shenzhen Zhongshan Urology Hospital), Shenzhen, 518000 People’s Republic of China; 3 Guangdong Engineering Technology Research Center of Reproductive Immunology for Peri-Implantation, Shenzhen, 518000 People’s Republic of China; 4 Shenzhen University Medical School, Shenzhen University, Shenzhen, 518000 People’s Republic of China; 5 Department of Obstetrics, People’s Hospital of Baoan, Shenzhen, 518000 People’s Republic of China; 6 Shenzhen Key Laboratory of Reproductive Immunology for Peri-Implantation, Guangdong Engineering Technology Research Center of Reproductive Immunology for Peri-Implantation, Shenzhen Zhongshan Obstetrics and Gynecology Hospital (Formerly Shenzhen Zhongshan Urology Hospital), Fuqiang Avenue 1001, Shenzhen, 518060 Guangdong People’s Republic of China

**Keywords:** Mesenchymal stem/stromal cells, MSCs, TSG6, CD317, Immunosuppression

## Abstract

**Background:**

Although both preclinical and clinical studies have shown the great application potential of MSCs (mesenchymal stem/stromal cells) in treating many kinds of diseases, therapeutic inconsistency resulting from cell heterogeneity is the major stumbling block to their clinical applications. Cell population diversity and batch variation in the cell expansion medium are two major inducers of MSC heterogeneity.

**Methods:**

Cell population diversity was investigated through single-cell RNA sequencing analysis of human MSCs derived from the umbilical cord and expanded with fully chemically defined medium in the current study. Then, the MSC subpopulation with enhanced anti-inflammatory effects was studied in vitro and in vivo.

**Results:**

Our data showed that MSCs contain different populations with different functions, including subpopulations with enhanced functions of exosome secretion, extracellular matrix modification and responses to stimuli (regeneration and immune response). Among them, CD317^+^ MSCs have improved differentiation capabilities and enhanced immune suppression activities. Underlying mechanism studies showed that higher levels of TSG6 confer enhanced anti-inflammatory functions of CD317^+^ MSCs.

**Conclusions:**

Thus, CD317^+^ MSCs might be a promising candidate for treating immunological disorder-related diseases.

**Supplementary Information:**

The online version contains supplementary material available at 10.1186/s13287-023-03618-8.

## Background

Mesenchymal stem/stromal cells (MSCs) have been intensively and extensively investigated in both preclinical and clinical studies. They have shown promising potential in treating many kinds of diseases through their microenvironment-modulating functions, such as immune modulation and regenerative functions [1–5]. Although clinical trials of MSCs have grown rapidly in recent years, few of them have achieved expected clinical outcomes, mainly resulting from the heterogeneity of MSCs [1, 4, 6].

MSC heterogeneity and therapeutic inconsistency severely hamper their clinical applications [1, 2, 7]. Sourcing, handling, healthy conditions and the genetic backgrounds of donors could induce heterogeneity [1, 7]. Many efforts have been made to address the issue of MSC heterogeneity [1, 6], such as genetic modification and expansion with chemically defined media [8–10]. It is suggested that homogenous MSC populations might yield more consistent clinical outcomes [6]. Thus, purifying specific MSC subpopulations for specific therapeutic purposes is another promising approach to reduce the heterogeneity of MSCs and improve their therapeutic consistency [1, 4]. Some human MSC markers have been identified, such as Stro-1 [11], CD166 [12], CD271 [13], CXCR4 [14], GD2 [15], CD146 [16], CD200 [17], CD49f, PODXL [18], CD140α [19], Lgr6 [20], Lgr5 [20], ROR2 [21], CD264 [22], CD143 [23] and CD362 [24]. However, these identified MSC markers are not MSC-specific. Furthermore, the underlying mechanisms of these MSC markers on their functions remain largely unsolved. Therefore, more MSC-specific markers need to be identified.

Cell subpopulation identification, based on transcriptome diversity revealed via high-throughput single-cell RNA sequencing (scRNA-seq), makes uncovering new MSC markers possible [25–30]. Unfortunately, few of these studies have identified novel MSC markers. Previously, we found that the conventional MSC expansion strategy with human platelet lysate induces MSC heterogeneity, and MSCs expanded with chemically defined medium have shown improved therapeutic consistency [9]. In addition, we have developed a fully chemically defined medium for expanding human MSCs without losing their characteristics and functions [9, 10]. Therefore, the aim of this study was to uncover the MSC heterogeneity resulting from cell population diversity and batch variation in cell expansion medium by scRNA-seq analysis and to identify the MSC subpopulation with enhanced immune suppression activities and therapeutic effects in a mouse model of acute inflammation.

## Methods

### Human MSC isolation, expansion and characterization

This study was approved by the ethics committee of Shenzhen Zhongshan Obstetrics & Gynecology Hospital (formerly Shenzhen Zhongshan Urology Hospital) and followed the tenants of the Declaration of Helsinki. Human MSCs were derived from the umbilical cord as described previously [8–10, 31]. Briefly, the human umbilical cords were minced, digested with 1 mg/mL collagenase B (STEMCELL Technologies) and expanded with the chemically defined medium NBVbe [10]. Human MSCs were passaged with TrypLE (Thermo Scientific) and stimulated with 20 ng/ml IFN-γ (PeproTech). MSC differentiation and characterization were performed with a StemPro^®^ Adipogenesis Differentiation Kit (Gibco), StemPro^®^ Osteogenesis Differentiation Kit (Gibco) and StemPro^®^ Chondrogenesis Differentiation Kit (Gibco) as described previously [10].

### Single-cell RNA-seq and analysis

Human MSCs derived from the umbilical cord and expanded with chemically defined medium [10] were prepared for scRNA-seq (single-cell RNA sequencing) at passage 3 as described previously [31]. Briefly, the MSCs were detached with TrypLE and resuspended in 0.04% BSA in HBSS (1 × 10^6^ cells/mL). The libraries were constructed with a 10 × Genomics Chromium platform and sequenced with an Illumina NovaSeq 6000 System (paired-end mode). Data were processed with the 10 × Genomics pipeline Cell Ranger (v2.1.0) and analyzed with the Seurat package in R (v 4.0.0).

### Flow cytometry

Cell preparation, antibody staining and flow cytometry were performed as described previously [8–10, 31]. Briefly, the MSCs were detached with TrypLE, resuspended in PBS plus 5% BSA (bovine serum albumin, Sigma) and incubated with anti-CD317-PE (Thermo Fisher Scientific), anti-CD73-FITC, anti-CD90-FITC, anti-CD105-FITC, anti-CD45-FITC, anti-CD34-FITC, anti-CD19-FITC, anti-CD11b-FITC, anti-HLADR-FITC, IgG-PE or IgG-FITC (all from BD Biosciences). Data were collected with BD AccuriC6 Plus (BD Biosciences) and analyzed with FlowJo software.

### ***CD317***^+^***/CD317***^***−***^*** MSC purification***

Human MSCs derived from the umbilical cord and expanded with chemically defined medium [10] were prepared for cell purification at passage 3. The MSCs were detached with TrypLE and stained with anti-CD317-PE (Thermo Fisher Scientific) or IgG-PE. Then, the CD317^+^ and CD317^−^ MSCs were purified with the BD FACSAria SORP cell sorter (BD Biosciences). Total RNA sequencing was performed at BGI (Beijing Genomics Institute) as described previously [10]. For shRNA construction, target sequences were cloned and inserted into the lentivirus pLKO.1-puro vector as described previously [32]. Lentivirus production and cell infection were performed as described previously [8]. Target sequences are listed in Additional file 1: Table S1.

### ELISA and qPCR

The CD317^+^ or CD317^−^ MSCs were plated onto 12-well plates (20 × 10^4^ cells per well), and the cell culture supernatant was collected three days later. The protein levels of CCL2 and TSG6 were measured with a Human MCP-1/CCL2 ELISA Kit (Sigma) and Human TSG6 ELISA Kit (Thermo Fisher Scientific) according to the instructions.

Peripheral blood was collected from the eyes of the mice, and the serum levels of IL-6 (BioLegend), TNF-α (BioLegend), IFN-γ (BioLegend) and IL-1β (BioLegend) were measured with ELISA kits as described previously [31]. Quantitative PCR (qPCR) was performed as described before after total RNA extraction and reverse transcription [8, 31]. The primer sequences are listed in Additional file 1: Table S1.

### MSC-PBMC coculture

Whole blood was collected into 10-mL EDTA tubes from 8 healthy subjects. Written informed consent was received from donors prior to the study. This study was approved by the ethics committee of Shenzhen Zhongshan Obstetrics & Gynecology Hospital (formerly Shenzhen Zhongshan Urology Hospital) and followed the tenants of the Declaration of Helsinki. Human PBMCs (peripheral blood mononuclear cells) were purified with the EasySep™ Direct Human PBMC Isolation Kit (STEMCELL Technologies). MSC-PBMC coculture was performed as described previously with modifications [8, 31]. Briefly, PBMCs were stimulated with Dynabeads^®^ Human T-Activator CD3/CD28 (Thermo Fisher Scientific) for 24 h and then cocultured with purified CD317^+^ or CD317^−^ MSCs (20 × 10^4^ PBMCs vs. 5 × 10^4^ MSCs) for 72 h. Cell proliferation was assessed with the Cell Proliferation Kit I (Roche) and quantified by an automated microplate reader (Bio-Rad) at 570 nm.

### Cell proliferation analysis

Cell proliferation was assessed as described previously [10]. Briefly, CD317^+^ and CD317^−^ MSCs were purified with FACS and plated onto p6 plates at a concentration of 10 × 10^4^ cells per well. When the cell confluence reached 80–90%, the MSCs were detached with TrypLE and counted with a hemocytometer, and the dead cells were identified with a cytotoxicity detection kit (Sigma).

### Mouse model of acute inflammation and cell transplantation

The mice (C57BL/6 J, female, 8 weeks old) were purchased from the Guangdong Medical Laboratory Animal Center and maintained in specific pathogen-free conditions. This study adheres to the ARRIVE guidelines and was approved by the Animal Research Ethics Committee of the School of Medicine, Shenzhen University. Mice were divided into 5 groups of eight mice each as follows: Group I, mice transplanted with PBS; Group II, mice transplanted with CD317^+^ MSCs; Group III, mice transplanted with CD317^−^ MSCs; Group IV, mice transplanted with CD317^+^ MSCs infected with lentivirus expressing scramble shRNA (negative control for shRNA experiment); and Group V, mice transplanted with CD317^+^ MSCs infected with lentivirus expressing shRNA targeting TSG6.

The mouse model of acute inflammation was induced by endotoxin LPS (lipopolysaccharides) as described [31]. Briefly, LPS was intraperitoneally injected into the mice (20 mg/kg, Sigma). Ten minutes later, PBS, CD317^+^ MSCs or CD317^−^ MSCs were intraperitoneally transplanted into the mouse model (1 × 10^6^ cells/mouse).

### Lung analysis

Mice were anesthetized with isoflurane by using the anesthesia system (R550, RWD Life Science) and euthanized with overdose CO_2_. The analysis of immune cell infiltration and MPO (myeloperoxidase) activities was performed as described [31]. Briefly, CD45^+^ lymphocytes and neutrophils (CD45^+^CD11b^+^Ly-6G^+^Ly-6C^med^) in BAL (bronchoalveolar lavage) were measured by flow cytometry. The MPO activity was determined by the MPO Activity Assay Kit (Abcam). HE (hematoxylin and eosin) staining of the lung tissue was performed as described [8, 31].

### Statistics

Data are shown as the mean ± SEM (standard error of the mean) and were analyzed with SPSS software for Windows (SPSS Inc.). Student’s *t* test was applied to the two-group comparison. One-way ANOVA was applied to the multiple group comparison with normal data distribution, parametric test and Tukey post hoc tests. *P* < 0.05 indicates statistical significance.

## Results

Our previous investigations have shown that conventional culture medium containing hPL (human platelet lysate) could induce MSC heterogeneity and therapeutic inconsistency [9]. Interestingly, MSCs expanded with chemically defined medium have reduced heterogeneity and improved the therapeutic efficacy and consistency of MSCs [9]. Furthermore, the distribution and constitution of MSC subpopulations varied significantly among MSCs expanded with different batches of hPL cells, which was revealed through scRNA-seq analysis, while they were more stable in chemically defined medium (data not shown, unpublished).

**Fig. 1:**
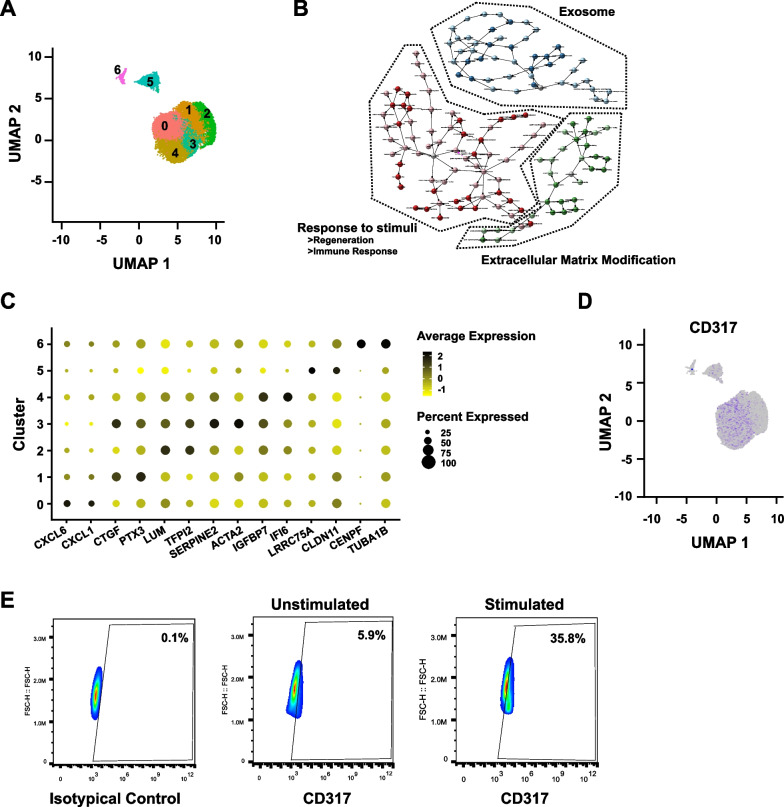
Identification of CD317^+^ MSCs. **A** Cell cluster identification via nonlinear dimensionality reduction analysis with UMAP. **B** Biological function clustering based on KEGG and GO network analysis. **C** Expression levels of the top 2 markers among different clusters. **D** Plotting of CD317 among different MSC clusters. **E** Flow cytometry analysis of CD317 expression without or with 20 ng/ml IFN-γ for 48 h. UMAP, uniform manifold approximation and projection; KEGG, Kyoto Encyclopedia of Genes and Genomes; GO, gene ontology; IFN-γ, interferon gamma

Therefore, to identify the potential cell markers of human MSCs expanded with chemically defined medium, which might have improved therapeutic efficacy and consistency, scRNA-seq was performed in human MSCs derived from the umbilical cord. A total of 888,476,328 reads were detected, 95.30% of which were valid barcodes (Additional file 1: Table S2, Fig. S1). The estimated number of cells was 12,760 in total (Additional file 1: Table S2). There were 12,063 genes detected in total, and the median number of genes per cell was 4,916 (Additional file 1: Table S2). Nonlinear dimensionality reduction analysis with UMAP (Uniform Manifold Approximation and Projection) showed that 7 different clusters were detected (Fig. 1A, Additional file 1: Fig. S2). DEG (differentially expressed gene) analysis revealed that these 7 MSC clusters had different transcriptomes (Additional file 1: Table S3). KEGG and GO analyses showed that the MSCs clustered into 3 different groups based on their biological functions, including the subpopulations with enhanced functions of exosome secretion, extracellular matrix modification and responses to stimuli (regeneration and immune response) (Fig. 1B), which is in accordance with the current understanding of MSC functions [1–5].

Marker gene identification indicated that different clusters predominantly expressed a panel of potential markers (Fig. 1C, Additional file 1: Table S4). Unfortunately, these bioinformatic identified potential markers could not discriminate different clusters clearly (Fig. 1C). They had different expression levels among different clusters, and different percentages of cells were positive within the clusters (Fig. 1C). It is well known that MSCs have high levels of plasticity [33]. Therefore, it is possible that the bioinformatic identified markers could not discriminate different clusters [31]. We also tried to adjust the parameters to reduce or increase the cluster numbers of the MSCs. However, similar results were obtained (data not shown). The different functions of MSCs revealed by the bioinformatic analysis prompted us to try alternative strategies to uncover different MSC populations with different functions (Fig. 1B). Thus, the mRNA levels of all identified cell membrane proteins were plotted. Our data showed that CD317 was predominantly expressed within some MSCs but not others (Fig. 1D). Its expression levels could be further induced by IFN-γ (Fig. 1E), which is in accordance with previous findings [34]. Thus, CD317 is a potential cell surface marker for labeling MSCs, which were purified from the human umbilical cord and expanded with chemically defined medium.

**Fig. 2:**
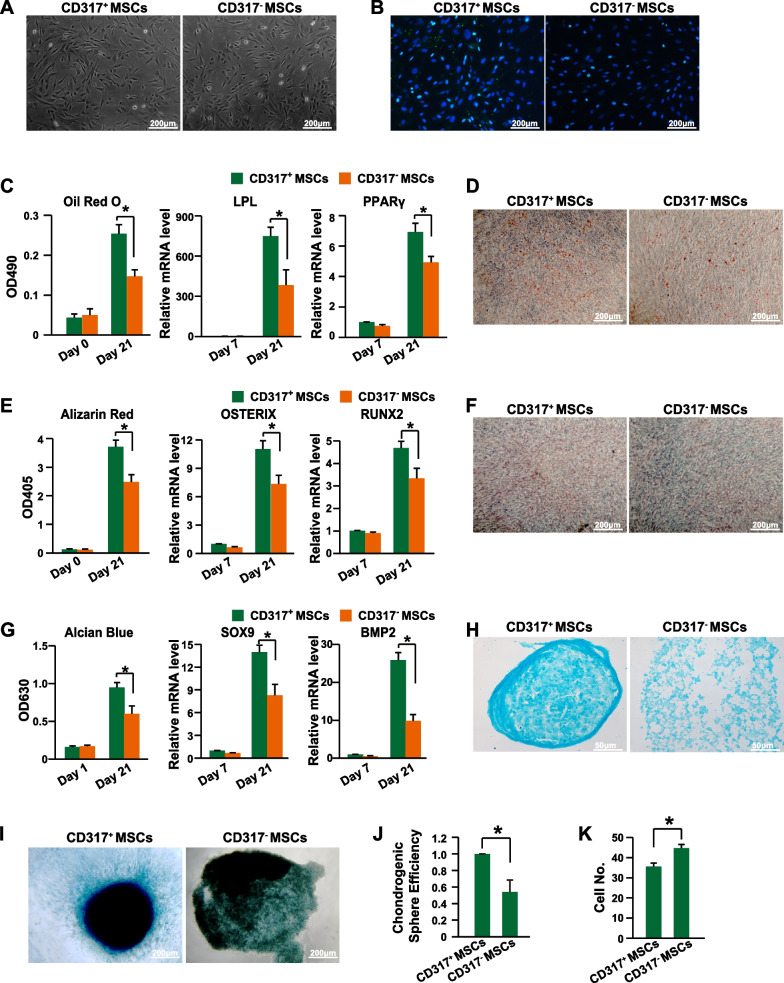
Characterization of CD317^+^ MSCs. **A** Cell morphology of CD317^+^ and CD317^−^ MSCs. **B** Immunofluorescence analysis of CD317 expression in CD317^+^ and CD317^−^ MSCs. **C** The adipocyte differentiation efficiency was quantified by Oil Red O staining and qPCR analysis of the LPL and PPARγ genes (*n *= 3). **D** Representative images of adipocyte differentiation stained with Oil Red O. **E** The osteocyte differentiation efficiency was quantified by Alizarin Red staining and qPCR analysis of the OSTERIX and RUNX2 genes (*n *= 3). **F** Representative images of osteocyte differentiation stained with Alizarin Red. **G** Chondrocyte differentiation efficiency was quantified by Alcian blue staining and qPCR analysis of the genes SOX9 and BMP2 (*n *= 3). **H** Representative images of chondrocyte differentiation stained with Alcian blue after sectioning. **I** Representative sphere images of chondrocyte differentiation stained with Alcian blue. **J** Chondrogenic sphere forming efficiency analysis (*n *= 3). **K** Cell proliferation of MSCs was determined by cell number counting (*n *= 3). MSCs, human mesenchymal stem/stromal cells; LPL, lipoprotein lipase; PPARγ, peroxisome proliferator activated receptor gamma; OSTERIX, Sp7 transcription factor; RUNX2, RUNX family transcription Factor 2; SOX9, SRY-box transcription Factor 9; BMP2, bone morphogenetic protein 2. * indicates *P *< 0.05

To further characterize the functions of CD317^+^ MSCs, they were purified with FACS (fluorescence-activated cell sorting). The CD317^+^ MSCs and CD317^−^ MSCs showed similar morphology (Fig. 2A) and levels of MSC marker expression (Additional file 1: Fig. S3). CD317 expression was detected in CD317^+^ MSCs but not CD317^−^ MSCs by immunofluorescence analysis (Fig. 2B). A tri-differentiation assay showed that CD317^+^ MSCs had a higher efficiency of differentiating into adipocytes (Fig. 2C, D), osteocytes (Fig. 2E, F) and chondrocytes (Fig. 2G–J). Furthermore, the CD317^+^ MSCs had a slower proliferation rate than the CD317^−^ MSCs (Fig. 2K). Therefore, CD317^+^ MSCs have classical MSC characteristics, better tri-differentiation efficiency and a slower proliferation rate.

**Fig. 3:**
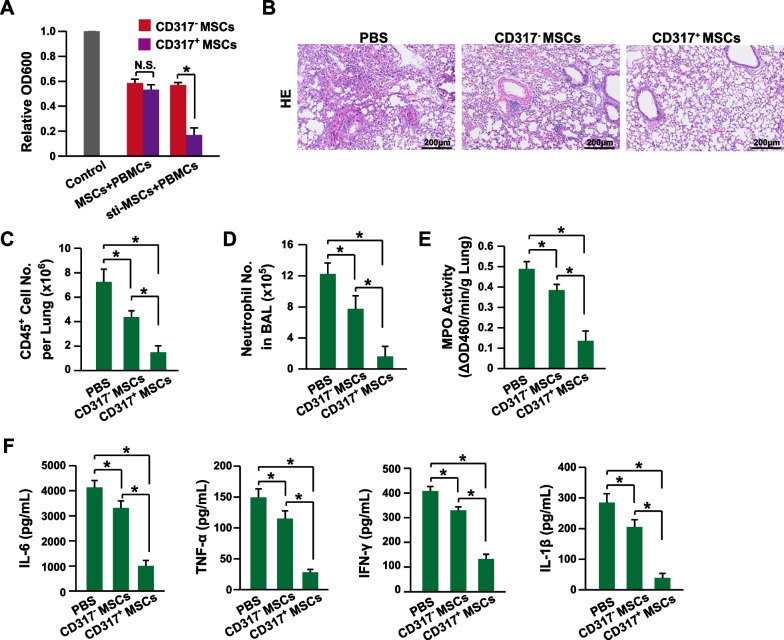
Enhanced immune suppression of CD317^+^ MSCs. **A** PBMC proliferation assay after coculture with CD317^+^ or CD317^−^ MSCs without or with 20 ng/ml IFN-γ for 48 h (*n *= 3). **B** Representative images of HE staining of lung tissues 24 h after LPS stimulation. **C** The CD45^+^ cells in the lung were measured 24 h after LPS stimulation via flow cytometry (*n *= 8). **D** The neutrophil number in BAL fluid was determined as CD45^+^CD11b^+^Ly-6G^+^Ly-6C^med^ 24 h after LPS stimulation by flow cytometry (*n *= 8). **E** MPO activity was quantified 24 h after LPS stimulation (*n *= 8). (**F**) Serum levels of IL-6, TNF-α, IFN-γ and IL-1β were determined 24 h after LPS stimulation via ELISA (*n *= 8). MSCs, human mesenchymal stem/stromal cells; sti-MSCs, MSCs stimulated with 20 ng/ml IFN-γ for 48 h; PBMCs, peripheral blood mononuclear cells; HE, hematoxylin and eosin; BAL, bronchoalveolar lavage; MPO, myeloperoxidase; IL-6, interleukin 6; TNF-α, tumor necrosis factor alpha; IFN-γ, interferon gamma; IL-1β, interleukin 1 beta. * indicates *P* < 0.05

Then, the immune suppression activities between the CD317^+^ MSCs and CD317^−^ MSCs were estimated by MSC-PBMC coculture. The data showed that both types of MSCs had similar levels of suppressing lymphocyte proliferation (Fig. 3A). However, after simulation with IFN-γ, the CD317^+^ MSCs had significantly higher suppression activities (Fig. 3A). To further confirm the stronger immune suppression activity of CD317^+^ MSCs in vivo, an LPS-induced mouse model of acute inflammation was established. Indeed, CD317^+^ MSCs maintained the tissue structure (Fig. 3B) and reduced CD45^+^ lymphocyte infiltration (Fig. 3C) more significantly than CD317^−^ MSCs. Furthermore, CD317^+^ MSCs reduced neutrophil infiltration (Fig. 3D, E) and the serum levels of proinflammatory cytokines (IL-6, TNF-α, IFN-γ and IL-1β) (Fig. 3F) more significantly than CD317^−^ MSCs. Thus, CD317^+^ MSCs had stronger immune suppression activity than CD317^−^ MSCs both in vitro and in vivo.

**Fig. 4:**
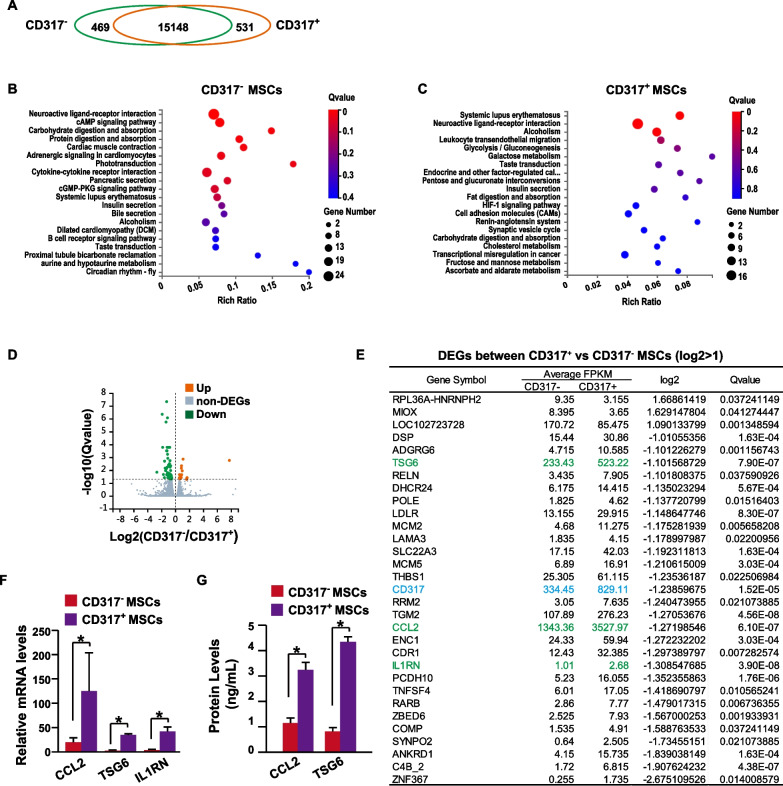
Transcriptome analysis CD317^+^ and CD317^−^ MSCs. **A** Venn diagram showing the numbers of genes differentially expressed in CD317^+^ and CD317^−^ MSCs. **B** GO enrichment analysis of genes specifically expressed in CD317^−^ MSCs. **C** GO enrichment analysis of genes specifically expressed in CD317^+^ MSCs. **D** Differentially expressed gene analysis between CD317^+^ and CD317^−^ MSCs. **E** Differentially expressed genes between CD317^+^ and CD317^−^ hMSCs with log2 > 1. **F** The mRNA levels of CCL2, TSG6 and IL1RN were determined via qPCR after stimulation with 20 ng/mL IFN-γ for 48 h (*n *= 3). **G** The protein levels of CCL2 and TSG6 were determined via ELISA after stimulation with 20 ng/mL IFN-γ for 48 h (*n *= 3). MSCs, human mesenchymal stem/stromal cells; DEGs, differentially expressed genes; CCL2, C–C motif chemokine ligand 2; TSG6, tumor necrosis factor-stimulated gene-6; IL1RN, interleukin 1 receptor antagonist; IFN-γ, interferon gamma. * indicates *P* < 0.05

CD317, also known as tetherin or BST2 (bone marrow stromal cell antigen 2), is involved in virus production and immune modulation [35]. However, the functions and mechanisms of CD317 in MSCs are largely undetermined. To uncover the underlying mechanisms, the transcriptomes of CD317^+^ MSCs and CD317^−^ MSCs were analyzed (Additional file 1: Table S5). There were 469 genes specifically expressed in CD317^−^ MSCs, while 531 genes were specifically expressed in CD317^+^ MSCs (Fig. 4A). GO analysis showed that the CD317^−^ MSCs had functions such as ligand–receptor interaction, cAMP pathway, metabolism and cytoskeleton reorganization (Fig. 4B), while CD317^+^ MSCs had functions such as immune modulation, ligand–receptor interaction, migration and metabolism (Fig. 4C). Although both CD317^+^ MSCs and CD317^−^ MSCs had specifically expressed genes (Fig. 4A), their expression levels were quite low, with the highest FPKM (Fragments Per Kilobase of exon model per Million mapped fragments) values of 2.39 and 2.21, respectively (Additional file 1: Table S5). Therefore, we also analyzed the DEGs between CD317^+^ and CD317^−^ MSCs. Among 15,148 co-expressed genes, 77 genes were differentially expressed (Fig. 4D, Additional file 1: Table S5). There were 31 DEGs with log2 > 1 (Fig. 4E). Among these 31 genes, the TSG6 (tumor necrosis factor-stimulated gene-6), CCL2 (C–C motif chemokine ligand 2) and IL1RN (interleukin 1 receptor antagonist) genes are important immune regulators in MSCs [2]. Then, the mRNA levels of known anti-inflammatory cytokines in MSCs [2] were determined by qPCR with stimulation or without stimulation (Additional file 1: Fig. S4). The data revealed that the mRNA levels of CCL2, TSG6 and IL1RN were differentially expressed between CD317^+^ MSCs and CD317^−^ MSCs after stimulation with IFN-γ (Fig. 4F), which is in accordance with the RNA-seq data. This finding was further validated at the protein level (Fig. 4G). However, the protein level of IL1RN was too low to be detected, which might result from its low mRNA levels (Fig. 4E). Therefore, CCL2 and TSG6 might contribute to the stronger immune suppression activity of CD317^+^ MSCs.

**Fig. 5:**
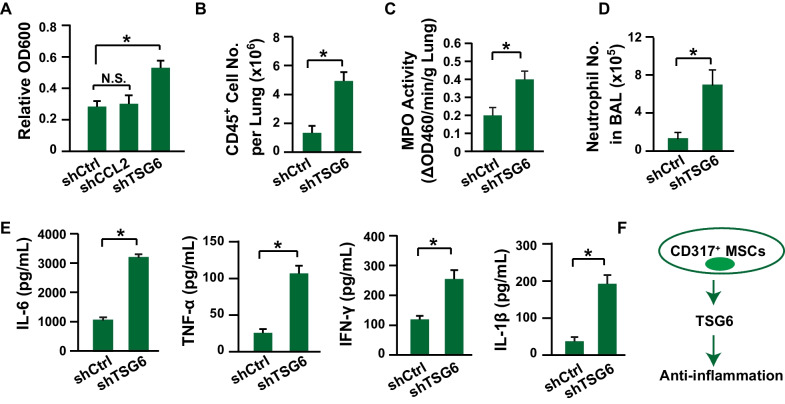
TSG6 contributes to the enhanced anti-inflammatory functions of CD317^+^ MSCs. **A** PBMC proliferation assay after coculture with MSCs (*n *= 3). **B** The CD45^+^ cells in the lung were measured 24 h after LPS stimulation via flow cytometry (*n *= 8). **C** MPO activity was quantified 24 h after LPS stimulation (*n *= 8). **D** The neutrophil number in BAL fluid was determined as CD45^+^CD11b^+^Ly-6G^+^Ly-6C^med^ 24 h after LPS stimulation by flow cytometry (*n *= 8). **E** Serum levels of IL-6, TNF-α, IFN-γ and IL-1β were determined 24 h after LPS stimulation via ELISA (*n *= 8). **F** Proposed potential mechanism of the enhanced immune suppression activities of CD317^+^ MSCs. MSCs, human mesenchymal stem/stromal cells stimulated with 20 ng/mL IFN-γ for 48 h; sh, knockdown of the corresponding gene with shRNA in CD317^+^ MSCs; TSG6, tumor necrosis factor-stimulated gene-6; BAL, bronchoalveolar lavage; MPO, myeloperoxidase; IL-6, interleukin 6; TNF-α, tumor necrosis factor alpha; IFN-γ, interferon gamma; IL-1β, interleukin 1 beta. * indicates *P *< 0.05

CCL2 is a major contributor to recruiting immune cells via the CCL2-CCR2 axis [36, 37]. Furthermore, it has been demonstrated that CCL2 expressed by MSCs can recruit monocytes and suppress inflammation [38]. TSG6, also known as TNFAIP6 (tumor necrosis factor alpha-induced protein 6), is a secreted glycoprotein with strong immune suppression activities [2, 39]. Our previous investigations have shown that TSG6^+^ mouse MSCs have improved immune suppression activities [31]. Therefore, TSG6 might be an important immune suppressor in CD317^+^ MSCs. Indeed, silencing TSG6 significantly impaired the immunosuppressive activity of CD317^+^ MSCs in vitro (Fig. 5A). Then, the function of TSG6 was further investigated in a mouse model of acute inflammation. Our data demonstrated that silencing TSG6 significantly impaired the immune suppression activities of CD317^+^ MSCs, from the perspectives of lymphocyte infiltration in the lung (Fig. 5B), MPO activity (Fig. 5C), neutrophil recruitment (Fig. 5D) and the serum levels of proinflammatory cytokines (Fig. 5E). Thus, TSG6 might confer the stronger immune suppression functions of CD317^+^ MSCs (Fig. 5J).

In summary, we have demonstrated here that the CD317^+^ subpopulation in human MSCs isolated from the umbilical cord and expanded with chemically defined medium has improved differentiation capabilities and enhanced immune suppression activities. The TSG6 secreted by MSCs might confer the enhanced immune suppression activities of CD317^+^ MSCs.

## Discussion

Although both preclinical and clinical studies have shown the great application potential of MSCs in treating many different kinds of diseases, the therapeutic inconsistency resulting from cell heterogeneity is the major stumbling block to their clinical applications [1–7]. We and other groups have made many efforts to reduce MSC heterogeneity and improve therapeutic efficacy and consistency [1, 6, 8–10]. Among different strategies, purifying homogenous MSC populations with enhanced biological functions is a promising approach [1, 4, 6].

Few human MSC-specific markers have been identified, although many different human MSC subpopulations have been demonstrated [11–24, 40]. Therefore, scRNA-seq has been applied to uncover new MSC markers and subpopulations [25–30]. Our previous investigations have demonstrated that the conventional MSC expansion strategy with human platelet lysate induces MSC heterogeneity, and MSCs expanded with chemically defined medium have shown improved therapeutic consistency [9]. Thus, we performed scRNA-seq analysis on human MSCs derived from the umbilical cord and expanded with fully chemically defined medium in the current study [9, 10].

Unfortunately, we also failed to uncover new human MSC markers via scRNA-seq analysis, which is in accordance with previous investigations [25–30]. The underlying mechanism might be the high levels of plasticity of MSCs [31, 33]. As our previous investigation also showed the limitations of bioinformatic analysis in identifying MSC markers [31], an alternative approach, in which the mRNA levels of genes expressed on the cell membrane were plotted on different clusters, was applied to identify new MSC markers. Among the three major MSC functions revealed by our scRNA-seq analysis, immune modulation is the most studied function in MSC biology and therapeutic applications. MSC marker identification analysis showed that CD317 is a potential MSC marker whose function is related to immune modulation [35]. Therefore, in the current study, we investigated the multipotent characteristics, immune suppression capability and underlying mechanism of CD317^+^ MSCs.

CD317, also known as tetherin or BST2 (bone marrow stromal cell antigen 2), is involved in virus production and immune modulation [35]. It has been demonstrated that CD317^+^ MSCs, which were derived from human bone marrow and immortalized, had higher levels of colony-forming capabilities with higher levels of IL-7 expression (40). Furthermore, the CD317^+^ circulating progenitors had higher regenerative potentials [41, 42]. However, whether our identified CD317^+^ MSCs, which were purified from the human umbilical cord and expanded with chemically defined medium, have similar functions to immortalized CD317^+^ MSCs isolated from bone marrow [40, 42] remains unclear.

The data here have shown that CD317^+^ human MSCs have better multipotency, from the perspectives of differentiating into adipocytes, osteocytes and chondrocytes; a slower proliferation rate; and enhanced immune suppression activities both in vitro and in vivo. However, Genever et al. demonstrated that CD317^+^ human MSCs, which were derived from bone marrow and immortalized, have reduced immune suppression activities and regenerative abilities [42]. The discrepancy might result from tissue origin (umbilical cord vs. bone marrow), expansion medium (chemically defined medium vs. fetal bovine serum) and cell population (primary vs. immortalized and clonal selected).

Transcriptome analysis showed that CD317^+^ MSCs express higher levels of TSG6. Both in vitro and in vivo studies showed that TSG6 contributes to the anti-inflammatory function of CD317^+^ MSCs. More interestingly, in the mouse model of acute inflammation induced by LPS, knocking down TSG6 could impair the immune suppression function of CD317^+^ MSCs. TSG6 is a secreted anti-inflammatory glycoprotein [39]. It has been demonstrated that TSG6 is a critical contributor to MSCs suppressing immune responses [43–57]. The expression level of TSG6 has been developed as a predictor of the therapeutic effects of MSCs in vivo [58]. Furthermore, we have identified that purified TSG6^+^ mouse MSCs have enhanced immune suppression activities and improved therapeutic effects in a mouse model of acute inflammation [31].

## Conclusions

In conclusion, we have identified that CD317^+^ MSCs have improved differentiation capabilities and enhanced immune suppression activities. Higher levels of TSG6 might confer the enhanced anti-inflammatory functions of CD317^+^ MSCs.

### Supplementary Information

**Additional file 1. **Supplementary Figures and Tables.

## Data Availability

The scRNA-seq dataset has been deposited into the China National Center for Bioinformation (https://www.cncb.ac.cn/) with the accession BioProject No. PRJCA021662.
