# Supplementary material for: CD317+ MSCs expanded with chemically defined media have enhanced immunological anti-inflammatory activities
Source: Stem Cell Res Ther. 2024 Jan 2;15:2. doi: 10.1186/s13287-023-03618-8 (PMC10763464; doi:10.1186/s13287-023-03618-8)
Supplement: Supplementary file 1 — Additional file 1. Supplementary Figures and Tables. [file 13287_2023_3618_MOESM1_ESM.pdf]

Supplementary Figure 1. Single cell RNA sequencing of hMSCs. (A) Number of feature RNA, count RNA, and the percentage of mitochondria genes before and after filtering. (**B**) ElbowPlot analysis showing the standard deviation of each PC. (**C**) Heatmaps of each PC.

Supplementary Figure 2. Potential marker gene expression in different clusters.

Supplementary Figure 3. MSC markers analysis. The expression levels of CD73, CD90, CD105, CD45, CD34, CD19, CD11b, and HLA-DR were determined by flow cytometry.

Supplementary Figure 4. Immune suppressor gene analysis. (A) The mRNA levels of IDO1, COX2, HLA-G, PD-L1, PD-L2, CD73, HO-1, Gal-9, CCL2, TSG6, and IL-1RN were determined via qPCR in $CD317^{-}$ and $CD317^{+}$ MSCs (n=3). **(B)** The mRNA levels of IDO1, COX2, HLA-G, PD-L1, PD-L2, CD73, HO-1, and Gal-9 were determined via qPCR in $CD317^{-}$ or $CD317^{+}$ MSCs and stimulated with 20ng/mL IFN-γ for 48 hours (n=3).

Supplementary Figure 1

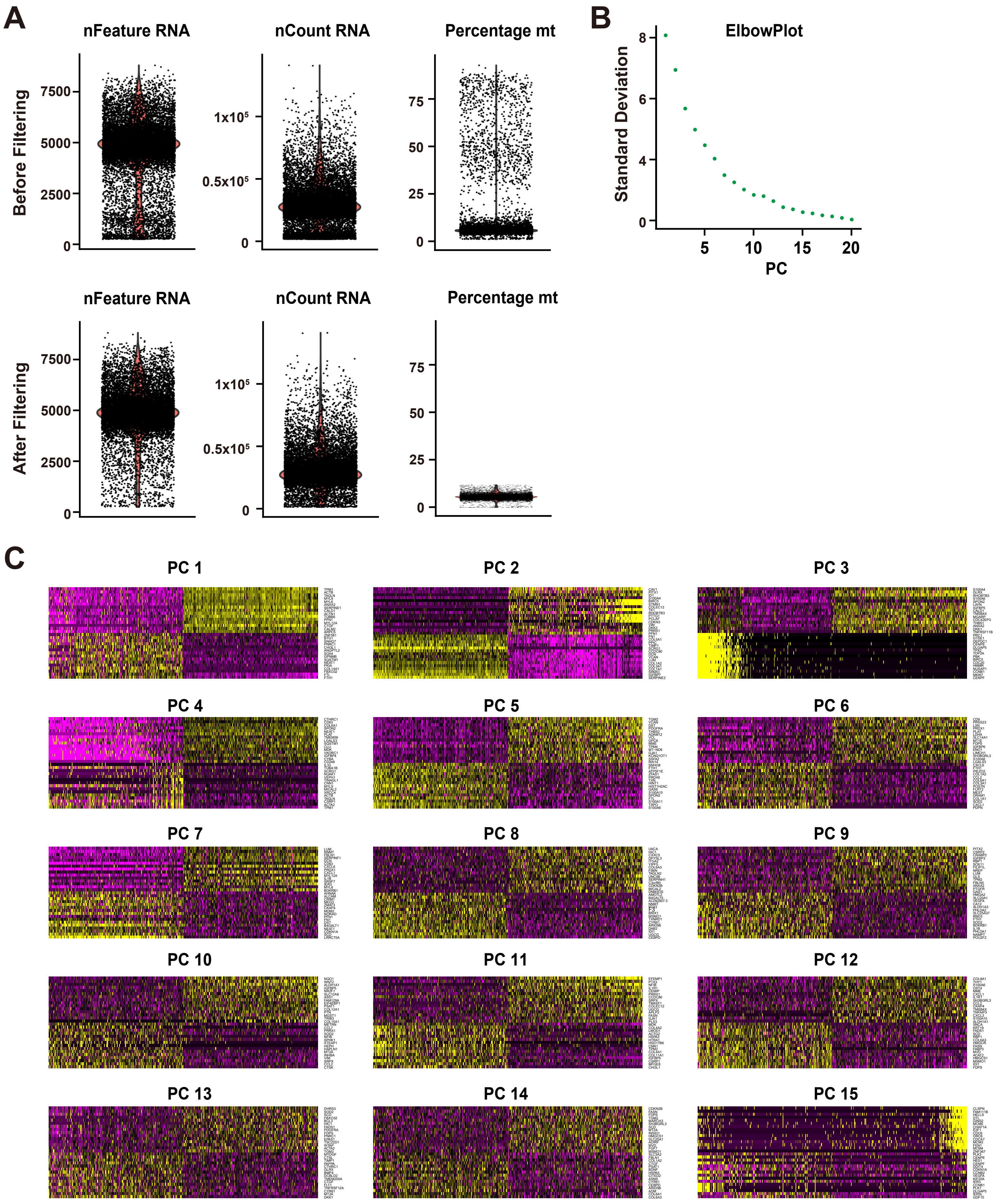

Supplementary Figure 2

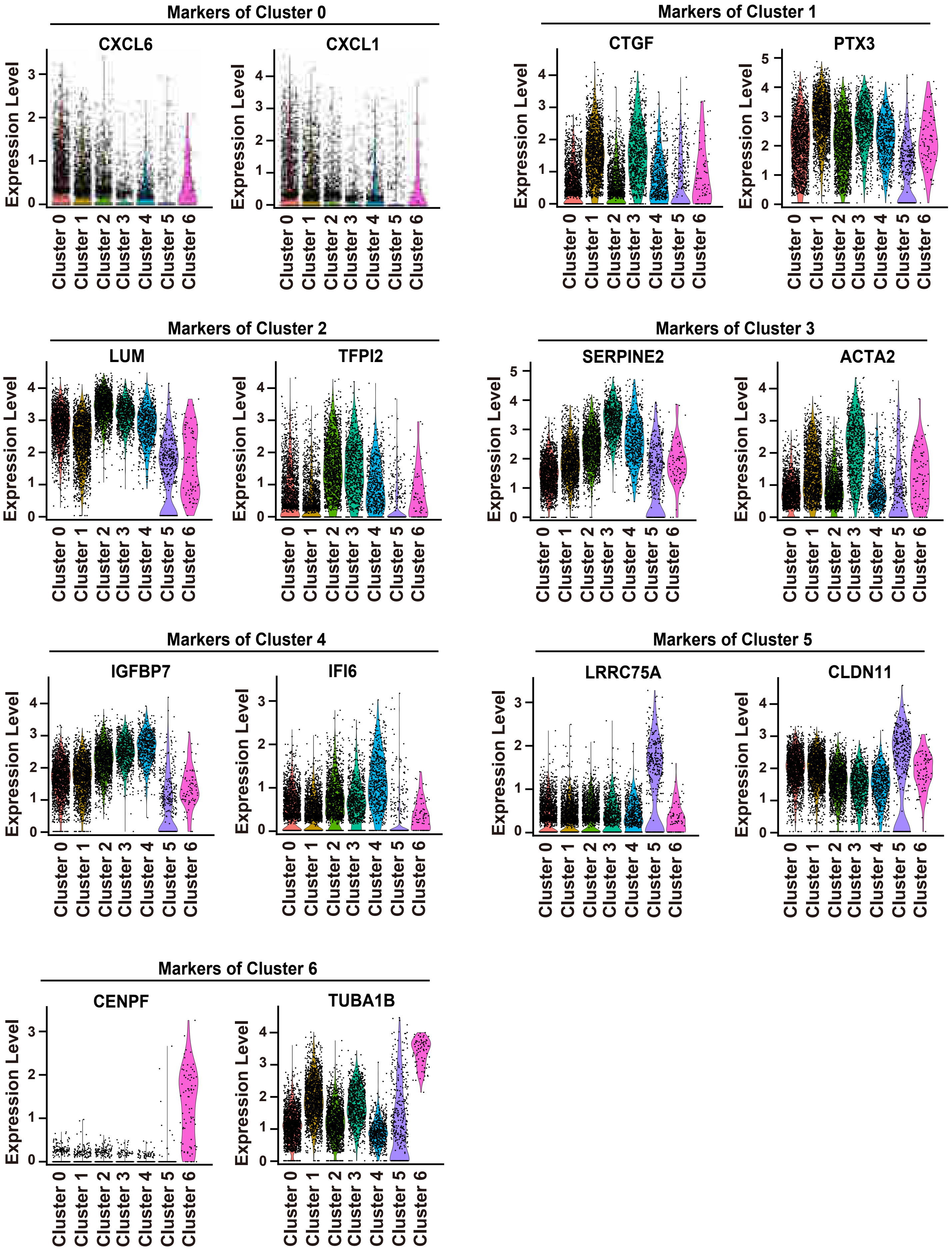

Supplementary Figure 3

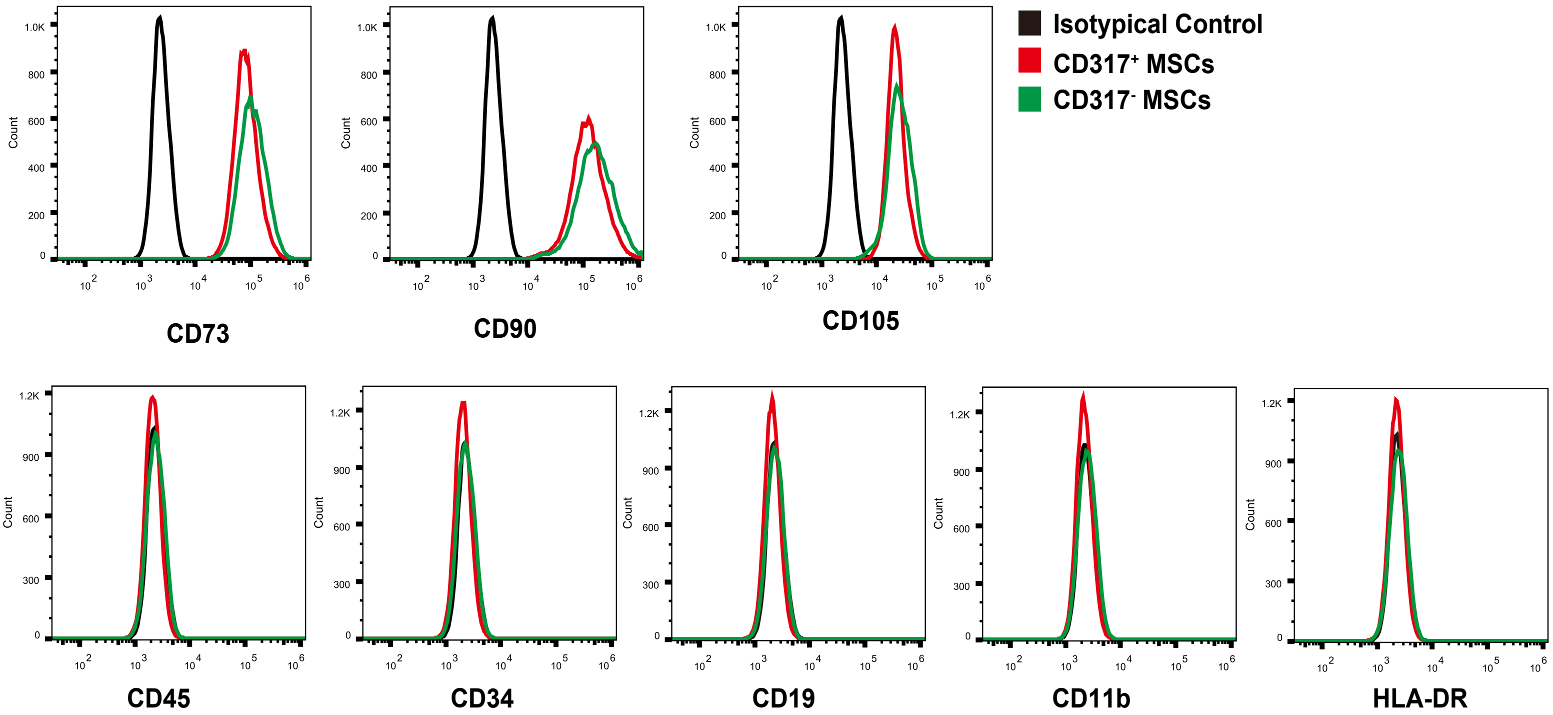

Supplementary Figure 4

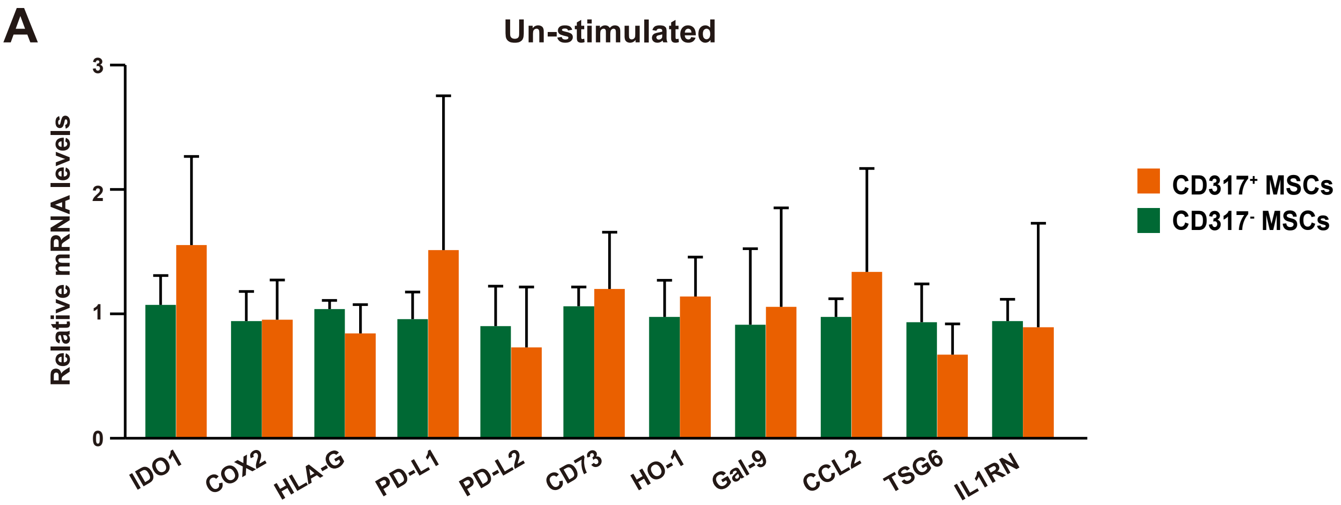

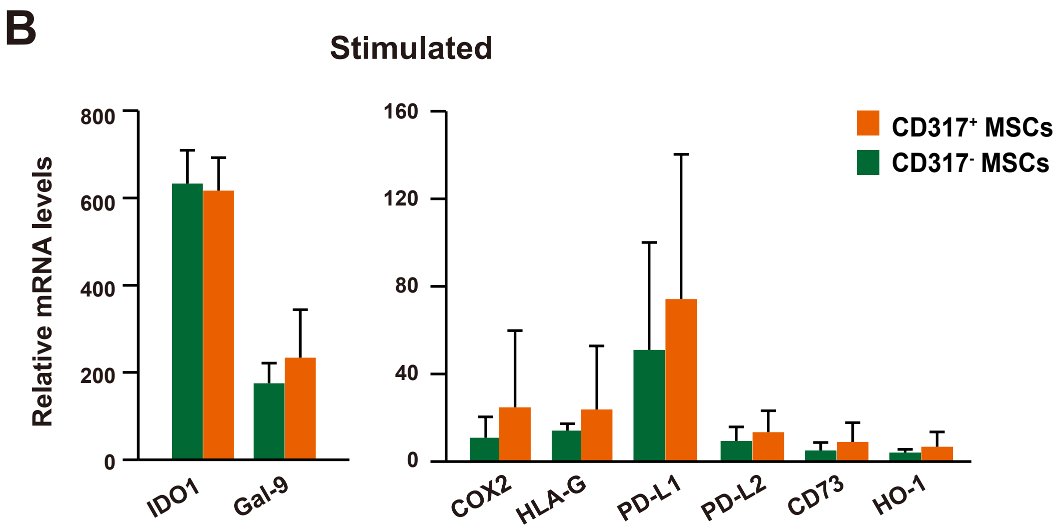

Supplementary Table 1. Primers and shRNAs

Name	Forwad 5`--3`	Reverse 5`--3`	Applications
IDO1	GCCTGATCTCATAGAGTCTGGC	TGCATCCCAGAACTAGACGTG	qPCR
COX2	CGGTGAAACTCTGGCTAGACAG	CAAACCGTAGATGCTCAGGGA	
HLA-G	GCGGCTACTACAACCAGAGC	GAGGTAATCCTTGCCATCGTAG	
PD-L1	GCCGACTACAAGCGAATTACTG	TGCTTGTCCAGATGACTTCGG	
PD-L2	CTCGTTCCACATACCTCAAGTCC	CTGGAACCTTTAGGATGTGAGTG	
CD73	TCCACTGGAGAGTTCCTGCA	TGAGAGGGTCATAACTGGGCA	
HO-1	CCAGGCAGAGAATGCTGAGT	AAGACTGGGCTCTCCTTGTTG	
Gal-9	ACACCCAGATCGACAACTCCT	CAAACAGGTGCTGACCATCCA	
CCL2	AGAATCACCAGCAGCAAGTGT	TCCTGAACCCACTTCTGCTTG	
TSG6	TCACCTACGCAGAAGCTAAGG	TCCAACTCTGCCCTTAGCCA	
IL-1RA	ATGGAGGGAAGATGTGCCTG	GTCCTGCTTTCTGTTCTCGCT	
LPL	CTGCTGGCATTGCAGGAAGTCT	CATCAGGAGAAAGACGACTCGG	
PPARG	AGCCTGCGAAAGCCTTTTGGTG	GGCTTCACATTCAGCAAACCTGG	
OSTERIX	TTCTGCGGCAAGAGGTTCACTC	GTGTTTGCTCAGGTGGTCGCTT	
RUNX2	CCCAGTATGAGAGTAGGTGTCC	GGGTAAGACTGGTCATAGGACC	
SOX9	AGGAAGCTCGCGGACCAGTAC	GGTGGTCCTTCTTGTGCTGCAC	
BMP2	TGTATCGCAGGCACTCAGGTCA	CCACTCGTTTCTGGTAGTTCTTC	
beta-actin	CACCATTGGCAATGAGCGG	AGGTCTTTGCGGATGTCCAC	
shCCL2	CCCAGTCACCTGCTGTTATAA		shRNA
shTSG6	GTGGCGTCTTTACAGATCCAA		
shCtrl	CCTAAGGTTAAGTCGCCCTCG		
shCD317	CCCAGGAAGCTGGCACATCTT		

SupplementaryTable 2. Seq-analysis inflomation

Table S2.1. Sequencing Statistics

Number of Reads	888,476,328
Valid Barcodes	95.30%
Valid UMIs	100.00%
Sequencing Saturation	31.90%
Q30 Bases in Barcode	98.30%
Q30 Bases in RNA Read	93.50%
Q30 Bases in Sample Index	96.20%
Q30 Bases in UMI	98.40%

Table S2.2. Mapping Statistics

Reads Mapped to Genome	95.80%
Reads Mapped Confidently to Genome	94.20%
Reads Mapped Confidently to Transcriptome	67.00%
Reads Mapped Confidently to Exonic Regions	71.70%
Reads Mapped Confidently to Intronic Regions	18.90%
Reads Mapped Confidently to Intergenic Regions	3.60%
Reads Mapped Antisense to Gene	1.60%

Table S2.3. Expression Statistics

Estimated Number of Cells	12,760
Fraction Reads in Cells	94.50%
Mean Reads per Cell	69,629
Median Genes per Cell	4,916
Total Genes Detected	23,063
Median UMI Counts per Cell	27,549

Supplementary Table 3. Differential expressed genes among different clusters

gene	p_val	avg_logF	pct.1	pct.2	p_val_adj	cluster
SPON2	3.69E-189	0.39714	0.996	0.939	6.57E-185	0
RAB13	1.15E-162	0.2508	0.999	0.978	2.05E-158	0
CTSK	9.05E-142	0.37391	0.768	0.543	1.61E-137	0
MDK	1.48E-137	0.34762	0.995	0.948	2.63E-133	0
VIM	3.42E-128	0.25897	1	1	6.08E-124	0
RBP1	7.67E-119	0.33416	0.993	0.941	1.37E-114	0
COL14A1	2.27E-116	0.28879	0.794	0.578	4.04E-112	0
POSTN	5.20E-114	0.45306	0.734	0.494	9.27E-110	0
TGFBI	7.54E-113	0.28878	1	0.981	1.34E-108	0
FBXO32	1.01E-109	0.27067	0.72	0.498	1.81E-105	0
LGALS3	1.10E-106	0.2582	0.998	0.954	1.96E-102	0
CXCL6	1.00E-105	0.47737	0.688	0.487	1.79E-101	0
PDGFRA	4.68E-105	0.25789	0.972	0.906	8.34E-101	0
SOX4	1.99E-104	0.29008	0.982	0.924	3.55E-100	0
FBLN1	1.90E-98	0.27216	0.992	0.945	3.38E-94	0
PLAT	1.31E-88	0.32638	0.966	0.876	2.33E-84	0
BDKRB1	5.09E-87	0.27364	0.798	0.642	9.07E-83	0
TMEM15	2.81E-81	0.32866	0.875	0.792	5.00E-77	0
CXCL1	1.67E-62	0.64776	0.592	0.433	2.98E-58	0
S100A4	1.84E-62	0.29834	0.958	0.857	3.28E-58	0
CHI3L1	5.40E-35	0.44729	0.628	0.509	9.61E-31	0
CCL2	7.94E-34	0.33313	0.926	0.881	1.41E-29	0
CXCL8	4.52E-31	0.30435	0.27	0.157	8.05E-27	0
RHOBTB	0	0.51053	1	0.966	0	1
ACTB	0	0.47239	1	0.998	0	1
ANXA2	0	0.44397	1	0.994	0	1
11-Sep	0	0.41865	0.998	0.951	0	1
MYL6	0	0.38502	1	0.991	0	1
CALD1	0	0.37546	1	0.993	0	1
CFL1	0	0.34963	1	0.988	0	1
TUBA1A	1.79E-297	0.51083	1	0.965	3.18E-293	1
TUBB6	2.85E-290	0.4083	0.988	0.868	5.07E-286	1
PFN1	1.23E-285	0.30584	1	0.99	2.18E-281	1
FLNA	4.15E-277	0.35721	0.999	0.966	7.40E-273	1
GLRX	1.05E-276	0.5234	0.998	0.935	1.86E-272	1
TUBA1B	5.29E-270	0.56529	0.997	0.955	9.42E-266	1
MYL12A	7.26E-267	0.33293	1	0.976	1.29E-262	1
COTL1	4.60E-260	0.34661	0.999	0.962	8.20E-256	1
ACTR3	1.47E-257	0.33257	0.998	0.926	2.61E-253	1
TPM1	3.63E-254	0.50801	0.999	0.991	6.46E-250	1
TPM3	8.14E-253	0.34918	0.998	0.949	1.45E-248	1
THBS1	1.42E-251	0.5453	0.964	0.777	2.53E-247	1
TPM4	9.49E-250	0.31493	1	0.99	1.69E-245	1
ARPC5	2.44E-235	0.29896	0.999	0.966	4.34E-231	1
CTGF	1.92E-234	0.69995	0.959	0.747	3.41E-230	1
ANXA1	2.02E-232	0.4592	0.999	0.971	3.59E-228	1
MYADM	4.58E-230	0.3532	0.964	0.783	8.15E-226	1
C12orf75	5.55E-226	0.37251	0.999	0.958	9.87E-222	1
MYL9	1.21E-225	0.36501	1	0.983	2.16E-221	1
TLN1	1.70E-224	0.29812	1	0.958	3.03E-220	1
CALM1	7.11E-223	0.29462	0.999	0.972	1.27E-218	1
CDC42EI	5.20E-222	0.3702	0.994	0.907	9.26E-218	1
CAP1	2.17E-217	0.29001	0.998	0.954	3.86E-213	1

CAV1	4.27E-211	0.60288	0.986	0.919	7.60E-207	1
LMO7	1.79E-210	0.34358	0.928	0.687	3.20E-206	1
ENO1	1.65E-208	0.319	1	0.972	2.94E-204	1
SRSF3	1.77E-208	0.3214	0.998	0.935	3.16E-204	1
ACTN4	7.51E-206	0.29295	0.98	0.879	1.34E-201	1
CYR61	1.71E-205	0.53575	0.972	0.817	3.04E-201	1
DKK1	3.04E-204	0.59021	0.912	0.665	5.41E-200	1
CRIM1	2.44E-201	0.30664	0.907	0.662	4.34E-197	1
PTX3	3.87E-198	0.68276	0.998	0.955	6.88E-194	1
MYH9	2.39E-195	0.30911	0.999	0.952	4.25E-191	1
PDLIM7	3.84E-191	0.2786	0.996	0.919	6.85E-187	1
TUBB4B	4.59E-191	0.28185	0.966	0.805	8.17E-187	1
CAVIN1	5.03E-191	0.26614	0.999	0.971	8.95E-187	1
OGFRL1	1.54E-190	0.31122	0.774	0.463	2.74E-186	1
S100A10	1.70E-187	0.29948	1	0.99	3.03E-183	1
ACTN1	2.72E-181	0.2778	0.998	0.957	4.85E-177	1
VCL	1.37E-177	0.29923	0.996	0.919	2.43E-173	1
CLIC1	3.27E-176	0.25339	1	0.973	5.83E-172	1
TAGLN	1.78E-175	0.38803	1	0.987	3.16E-171	1
TIMP3	1.02E-173	0.4476	0.973	0.841	1.81E-169	1
SRSF2	3.68E-171	0.25377	0.991	0.889	6.55E-167	1
LAYN	3.24E-167	0.2528	0.838	0.555	5.76E-163	1
RAI14	3.41E-162	0.27964	0.992	0.91	6.07E-158	1
TUBB2A	2.49E-161	0.27349	0.879	0.646	4.43E-157	1
ODC1	4.69E-160	0.26164	0.952	0.79	8.36E-156	1
NEXN	1.46E-155	0.27287	0.982	0.864	2.60E-151	1
TNFRSF1	1.88E-155	0.32937	0.992	0.911	3.34E-151	1
CDV3	1.17E-146	0.26209	0.993	0.943	2.08E-142	1
LMNA	6.04E-146	0.25914	1	0.962	1.07E-141	1
CITED2	3.56E-143	0.29167	0.874	0.668	6.34E-139	1
FERMT2	2.10E-141	0.25179	0.979	0.865	3.73E-137	1
PHLDA2	1.15E-140	0.29718	0.966	0.867	2.05E-136	1
MACF1	4.04E-140	0.25266	0.992	0.929	7.20E-136	1
TNFRSF1	7.70E-140	0.38251	0.661	0.361	1.37E-135	1
MAP1B	2.26E-138	0.26147	1	0.977	4.03E-134	1
TXNRD1	5.54E-138	0.3176	0.984	0.918	9.86E-134	1
CALM2	1.54E-134	0.25664	1	0.992	2.75E-130	1
PLS3	8.57E-127	0.26847	0.978	0.871	1.53E-122	1
ID3	1.31E-124	0.45728	0.887	0.689	2.33E-120	1
ID1	4.94E-122	0.39071	0.557	0.282	8.79E-118	1
NQO1	6.81E-121	0.28654	0.928	0.772	1.21E-116	1
BDNF	1.08E-119	0.26512	0.701	0.449	1.93E-115	1
F3	1.19E-111	0.25745	0.543	0.292	2.12E-107	1
CCND1	3.97E-106	0.25838	0.98	0.914	7.06E-102	1
DAB2	3.60E-99	0.25922	0.993	0.945	6.42E-95	1
KRT18	3.42E-97	0.4066	0.974	0.927	6.08E-93	1
GAS6	9.36E-96	0.26797	0.987	0.929	1.67E-91	1
S100A4	5.39E-92	0.38899	0.977	0.851	9.59E-88	1
SERPINE	7.39E-92	0.25553	0.973	0.861	1.32E-87	1
ACTG2	9.64E-83	0.27714	0.786	0.594	1.72E-78	1
KRT19	2.11E-76	0.38765	0.845	0.708	3.75E-72	1
CPA4	4.43E-76	0.26318	0.641	0.43	7.88E-72	1
LRRC17	3.84E-64	0.31877	0.8	0.685	6.83E-60	1
IGFBP5	1.07E-52	0.45233	0.96	0.915	1.91E-48	1
LUM	0	0.6424	1	0.978	0	2
DCN	7.47E-234	0.4976	1	0.98	1.33E-229	2

CTSL	1.46E-230	0.43191	0.993	0.935	2.59E-226	2
TFPI2	1.92E-212	0.74485	0.951	0.719	3.42E-208	2
HTRA1	1.07E-207	0.39763	0.956	0.755	1.91E-203	2
SERPINF	7.11E-189	0.38619	0.977	0.84	1.27E-184	2
B2M	2.66E-187	0.2802	1	0.992	4.74E-183	2
SLC14A1	7.73E-184	0.33108	0.635	0.286	1.38E-179	2
C1R	7.95E-162	0.29147	0.991	0.912	1.42E-157	2
MMP2	1.29E-153	0.27107	1	0.978	2.29E-149	2
VCAN	2.19E-148	0.34651	1	0.979	3.90E-144	2
C1S	2.09E-145	0.29455	0.983	0.883	3.73E-141	2
GNG11	8.13E-138	0.3443	0.988	0.935	1.45E-133	2
HNMT	1.43E-133	0.25203	0.763	0.495	2.54E-129	2
MFAP4	1.71E-131	0.26506	0.755	0.501	3.04E-127	2
TXNIP	7.39E-131	0.29823	0.977	0.876	1.32E-126	2
HLA-C	1.71E-130	0.28829	1	0.958	3.05E-126	2
IL33	3.21E-129	0.2695	0.378	0.126	5.72E-125	2
COL14A1	5.80E-126	0.313	0.839	0.574	1.03E-121	2
PLAC9	5.64E-108	0.25123	0.802	0.574	1.00E-103	2
SCRG1	1.04E-99	0.25078	0.737	0.466	1.85E-95	2
TIMP1	3.91E-99	0.2545	1	0.983	6.97E-95	2
TENT5A	8.66E-96	0.30505	0.941	0.839	1.54E-91	2
HLA-B	1.24E-94	0.29583	0.994	0.952	2.20E-90	2
RARRES	1.32E-91	0.271	0.8	0.639	2.36E-87	2
TNFAIP6	6.63E-89	0.27092	0.816	0.612	1.18E-84	2
LXN	3.56E-85	0.28607	0.953	0.856	6.34E-81	2
CLU	5.70E-85	0.35298	0.99	0.956	1.01E-80	2
IGFBP7	3.45E-84	0.26195	1	0.958	6.15E-80	2
FGF7	1.07E-71	0.28141	0.984	0.924	1.91E-67	2
CHI3L1	1.95E-68	0.62276	0.684	0.498	3.48E-64	2
MGP	2.62E-63	0.32006	0.493	0.293	4.67E-59	2
PLAT	1.37E-48	0.33526	0.944	0.886	2.44E-44	2
SERPINE	0	1.18668	1	0.974	0	3
TINAGL1	2.04E-279	0.26104	0.499	0.076	3.63E-275	3
PRSS23	4.66E-277	0.778	0.988	0.695	8.30E-273	3
TAGLN	1.44E-255	0.82778	1	0.989	2.57E-251	3
CCDC80	9.84E-235	0.77106	1	0.982	1.75E-230	3
TPM1	1.83E-227	0.77821	1	0.992	3.25E-223	3
SERPINE	6.02E-226	0.84714	0.998	0.875	1.07E-221	3
SCRG1	6.18E-217	0.69711	0.904	0.476	1.10E-212	3
ACTA2	9.63E-213	1.22859	0.992	0.919	1.71E-208	3
LIMCH1	2.22E-212	0.26545	0.595	0.148	3.95E-208	3
XRCC4	1.67E-186	0.73055	0.784	0.367	2.97E-182	3
THBS2	2.65E-175	0.38073	1	0.949	4.71E-171	3
MICAL2	2.44E-172	0.36522	0.892	0.525	4.34E-168	3
THY1	8.95E-172	0.45613	1	0.978	1.59E-167	3
VCAN	8.12E-167	0.50452	1	0.981	1.45E-162	3
CSRP1	6.94E-162	0.51141	0.994	0.887	1.24E-157	3
POMP	2.79E-154	0.31504	1	0.98	4.96E-150	3
INHBA	2.10E-152	0.59511	0.976	0.764	3.73E-148	3
ACTN1	4.60E-151	0.37832	1	0.963	8.20E-147	3
EFEMP1	6.35E-149	0.51575	0.929	0.609	1.13E-144	3
IGFBP7	6.01E-148	0.48451	0.999	0.963	1.07E-143	3
NUAK1	2.89E-145	0.34234	0.929	0.601	5.14E-141	3
CALU	1.47E-144	0.27143	1	0.977	2.61E-140	3
COL4A1	2.49E-140	0.49845	0.966	0.79	4.43E-136	3
FN1	2.96E-138	0.34998	1	0.991	5.27E-134	3

PDLIM3	1.26E-136	0.37592	0.917	0.614	2.24E-132	3
CALM2	1.20E-134	0.38604	1	0.994	2.13E-130	3
PLAC9	5.49E-133	0.33719	0.915	0.586	9.77E-129	3
SERPINH	1.62E-127	0.28231	1	0.977	2.88E-123	3
GLS	2.82E-126	0.38904	0.998	0.924	5.03E-122	3
TMEM16	3.21E-123	0.26874	1	0.968	5.72E-119	3
HAPLN1	3.63E-119	0.44535	0.628	0.282	6.47E-115	3
SPARC	3.75E-113	0.31457	1	0.987	6.68E-109	3
MYDGF	3.77E-113	0.28249	1	0.974	6.71E-109	3
LOX	1.20E-112	0.40927	1	0.96	2.14E-108	3
SULF1	2.63E-111	0.40225	0.964	0.749	4.68E-107	3
MYL12A	3.37E-110	0.28747	1	0.98	6.00E-106	3
TNFRSF1	5.63E-110	0.33863	0.999	0.923	1.00E-105	3
MYL9	1.13E-109	0.36493	1	0.986	2.02E-105	3
PAWR	1.77E-109	0.28662	0.937	0.704	3.15E-105	3
SMYD3	4.05E-108	0.29888	0.95	0.743	7.21E-104	3
PALLD	6.02E-107	0.36001	0.985	0.896	1.07E-102	3
FERMT2	2.06E-105	0.31377	0.994	0.881	3.67E-101	3
PRNP	4.00E-105	0.31054	0.931	0.687	7.12E-101	3
MTHFD2	8.51E-105	0.27948	0.992	0.908	1.51E-100	3
VEPH1	1.28E-104	0.27527	0.81	0.455	2.28E-100	3
COL5A1	1.55E-104	0.26583	1	0.974	2.77E-100	3
GPC6	1.08E-103	0.28451	0.949	0.713	1.93E-99	3
CDH2	1.27E-103	0.32453	0.999	0.962	2.26E-99	3
LMCD1	4.36E-103	0.35344	0.98	0.819	7.77E-99	3
NNMT	4.54E-102	0.2668	1	0.99	8.08E-98	3
PCDH10	2.61E-98	0.27447	0.841	0.523	4.64E-94	3
AMIGO2	1.34E-96	0.3303	0.855	0.551	2.39E-92	3
CTGF	4.56E-94	0.68939	0.959	0.78	8.11E-90	3
COL4A2	7.07E-92	0.33562	0.998	0.935	1.26E-87	3
ACTB	1.37E-90	0.28051	1	0.998	2.44E-86	3
PSAT1	1.90E-90	0.26712	0.987	0.894	3.38E-86	3
TFPI2	2.04E-90	0.53329	0.954	0.746	3.64E-86	3
S100A16	1.84E-89	0.26677	0.991	0.95	3.27E-85	3
FGF2	6.96E-88	0.29781	0.961	0.819	1.24E-83	3
PDLIM7	8.76E-87	0.25183	0.999	0.93	1.56E-82	3
DLC1	1.23E-86	0.25082	0.94	0.707	2.20E-82	3
TPM2	2.75E-84	0.25322	1	0.997	4.89E-80	3
COL11A1	5.53E-84	0.31407	0.836	0.599	9.85E-80	3
GADD45I	2.00E-83	0.25268	0.815	0.51	3.56E-79	3
CALD1	2.52E-82	0.26981	1	0.994	4.48E-78	3
KRT8	6.81E-82	0.37747	0.976	0.92	1.21E-77	3
FSTL3	3.08E-81	0.25912	0.864	0.607	5.48E-77	3
HTRA1	1.59E-79	0.26566	0.972	0.777	2.82E-75	3
LOXL1	1.30E-78	0.27164	0.923	0.751	2.32E-74	3
HIF1A	1.75E-75	0.25471	0.991	0.909	3.12E-71	3
TGM2	3.66E-71	0.39728	0.979	0.869	6.51E-67	3
MEST	1.99E-69	0.29547	1	0.947	3.54E-65	3
MYLK	3.21E-63	0.28403	0.775	0.533	5.72E-59	3
CEMIP	1.69E-56	0.31607	0.84	0.619	3.00E-52	3
ACTG2	5.57E-56	0.41672	0.825	0.618	9.91E-52	3
LUM	1.93E-55	0.26117	1	0.981	3.44E-51	3
KRT18	4.02E-51	0.34641	0.977	0.934	7.15E-47	3
DDIT4	1.08E-50	0.25172	0.974	0.875	1.92E-46	3
CYR61	1.48E-34	0.26797	0.943	0.844	2.64E-30	3
COL1A2	4.19E-166	0.38764	1	0.993	7.47E-162	4

SPARC	1.34E-164	0.51998	1	0.988	2.39E-160	4
LOX	3.80E-160	0.62753	1	0.961	6.77E-156	4
COL1A1	7.77E-152	0.3894	1	0.995	1.38E-147	4
IGFBP7	1.01E-151	0.63936	1	0.964	1.79E-147	4
ZFAS1	2.69E-148	0.39044	1	0.986	4.78E-144	4
HIST1H2	3.33E-139	0.30933	0.855	0.41	5.93E-135	4
FTL	1.63E-135	0.40569	1	1	2.90E-131	4
TIMP1	1.63E-126	0.6321	1	0.986	2.91E-122	4
IFI6	4.48E-120	0.66027	0.957	0.766	7.99E-116	4
PPIC	1.74E-116	0.35212	1	0.95	3.09E-112	4
FTH1	6.53E-115	0.36573	1	1	1.16E-110	4
TMEM59	2.28E-114	0.33735	1	0.962	4.05E-110	4
TSPO	2.31E-110	0.29429	1	0.986	4.12E-106	4
CCPG1	1.34E-109	0.37427	0.994	0.82	2.38E-105	4
SNHG8	1.76E-104	0.36811	1	0.977	3.13E-100	4
CST3	1.09E-99	0.25735	1	0.973	1.93E-95	4
CDKN1A	2.83E-90	0.45249	1	0.919	5.03E-86	4
P4HA2	4.04E-90	0.3076	0.998	0.945	7.20E-86	4
CD63	3.19E-83	0.25026	1	0.984	5.67E-79	4
COPZ2	4.39E-83	0.25938	1	0.967	7.81E-79	4
CYBA	1.17E-81	0.35419	0.965	0.918	2.09E-77	4
LOXL2	1.52E-80	0.27684	0.998	0.938	2.71E-76	4
DDIT3	1.49E-77	0.29676	0.987	0.866	2.65E-73	4
SPON2	8.69E-76	0.41228	0.994	0.949	1.55E-71	4
NDUFA4l	1.08E-70	0.41027	0.761	0.428	1.93E-66	4
GAS6	3.58E-70	0.36368	0.998	0.939	6.37E-66	4
MFGE8	4.60E-70	0.2999	0.991	0.914	8.19E-66	4
ASS1	7.77E-68	0.26352	0.957	0.722	1.38E-63	4
TIMP3	4.80E-65	0.57026	0.991	0.863	8.55E-61	4
CPA4	5.18E-65	0.3149	0.799	0.453	9.21E-61	4
MEST	7.68E-65	0.3947	0.998	0.949	1.37E-60	4
PTGDS	4.69E-64	0.27635	0.567	0.258	8.36E-60	4
GADD45/	5.65E-59	0.28943	0.975	0.802	1.01E-54	4
COX7A1	5.54E-56	0.28325	0.866	0.617	9.86E-52	4
MYDGF	1.07E-55	0.26063	1	0.975	1.90E-51	4
SERPINE	1.95E-54	0.49267	1	0.975	3.46E-50	4
THY1	4.24E-51	0.34571	1	0.979	7.54E-47	4
PAPPA	1.20E-45	0.25547	0.907	0.644	2.14E-41	4
SERPINE	4.28E-42	0.31923	0.989	0.879	7.62E-38	4
S100A10	4.57E-37	0.2583	1	0.992	8.14E-33	4
SCRG1	4.92E-37	0.38508	0.747	0.505	8.77E-33	4
GDF15	1.05E-36	0.3756	0.875	0.696	1.87E-32	4
XRCC4	2.33E-35	0.29278	0.635	0.394	4.14E-31	4
PABPC1	7.53E-45	0.39394	0.826	1	1.34E-40	5
LRRC75/	2.42E-39	1.10151	0.653	0.724	4.32E-35	5
SET	6.56E-21	0.35391	0.815	1	1.17E-16	5
HEXIM1	1.41E-19	0.27808	0.15	0.413	2.51E-15	5
TTC3	7.24E-17	0.3237	0.772	0.999	1.29E-12	5
PHKG1	7.32E-17	0.44925	0.399	0.308	1.30E-12	5
LPIN1	1.07E-15	0.25425	0.359	0.728	1.91E-11	5
RERE	1.17E-14	0.25746	0.347	0.702	2.08E-10	5
TPM4	3.18E-14	0.28726	0.876	1	5.66E-10	5
ZFP36L2	5.15E-14	0.30905	0.268	0.549	9.16E-10	5
BOLA3	1.04E-11	0.28156	0.42	0.803	1.85E-07	5
GLTP	1.79E-10	0.25017	0.329	0.645	3.19E-06	5
CLDN11	7.38E-10	0.53719	0.709	0.991	1.31E-05	5

NKTR	2.42E-09	0.26417	0.432	0.82	4.31E-05	5
YWHAE	1.96E-08	0.28375	0.777	0.999	0.000349093	5
PTP4A1	6.07E-08	0.26853	0.458	0.841	0.001080714	5
FTX	3.98E-07	0.29476	0.415	0.762	0.00708506	5
WSB1	4.41E-07	0.26735	0.556	0.961	0.007855963	5
PTP4A2	5.75E-07	0.25344	0.728	0.998	0.010244903	5
RBMS3	6.53E-07	0.26737	0.411	0.734	0.011623253	5
LASP1	1.06E-06	0.27489	0.477	0.842	0.018911048	5
DDAH1	2.67E-06	0.28305	0.296	0.526	0.047536457	5
TOP2A	0	1.36413	0.889	0.033	0	6
MKI67	0	1.01403	0.852	0.011	0	6
NUSAP1	0	0.92089	0.827	0.027	0	6
CDC20	0	0.91848	0.79	0.016	0	6
UBE2C	0	0.89947	0.691	0.017	0	6
DLGAP5	0	0.86911	0.753	0.006	0	6
HMMR	0	0.82068	0.84	0.011	0	6
PBK	0	0.69978	0.815	0.006	0	6
RRM2	0	0.69832	0.914	0.032	0	6
DEPDC1	0	0.63974	0.716	0.004	0	6
GTSE1	0	0.60798	0.741	0.007	0	6
CEP55	0	0.59639	0.765	0.006	0	6
PRC1	0	0.59147	0.815	0.018	0	6
ANLN	0	0.58327	0.716	0.011	0	6
NUF2	0	0.55993	0.617	0.006	0	6
KNL1	0	0.43691	0.605	0.011	0	6
KIF14	0	0.42142	0.543	0.006	0	6
CDCA8	0	0.39409	0.556	0.007	0	6
SGO1	0	0.38709	0.617	0.004	0	6
SPC25	0	0.38083	0.63	0.001	0	6
PIMREG	0	0.37754	0.58	0.002	0	6
KIF4A	0	0.37575	0.593	0.005	0	6
BUB1	0	0.34581	0.556	0.008	0	6
TTK	0	0.34248	0.543	0.007	0	6
BUB1B	0	0.33649	0.543	0.003	0	6
NEK2	0	0.33446	0.519	0.001	0	6
SHCBP1	0	0.33135	0.617	0.005	0	6
KIF20A	0	0.32107	0.58	0.004	0	6
MYBL2	0	0.3109	0.654	0.008	0	6
SPAG5	0	0.27012	0.531	0.005	0	6
NCAPG	1.62E-296	0.45726	0.704	0.021	2.89E-292	6
BIRC5	2.52E-293	1.20082	0.951	0.046	4.49E-289	6
CLSPN	1.89E-269	0.50216	0.654	0.021	3.36E-265	6
CDCA3	3.69E-249	0.49324	0.679	0.025	6.56E-245	6
CENPU	8.54E-238	0.28537	0.642	0.023	1.52E-233	6
KIF2C	2.82E-229	0.33928	0.556	0.017	5.01E-225	6
TACC3	3.49E-229	0.42574	0.728	0.033	6.21E-225	6
CENPE	3.59E-229	0.9013	0.716	0.032	6.38E-225	6
PLK1	1.56E-224	0.48671	0.568	0.019	2.79E-220	6
TRIP13	7.46E-217	0.27541	0.617	0.024	1.33E-212	6
CCNA2	3.57E-215	0.57998	0.679	0.03	6.36E-211	6
CENPA	4.73E-203	0.47275	0.58	0.022	8.43E-199	6
CDKN3	1.78E-198	0.94548	0.889	0.063	3.17E-194	6
CENPF	3.54E-198	1.86088	0.914	0.068	6.30E-194	6
KIF11	6.30E-195	0.44474	0.679	0.034	1.12E-190	6
NDC80	1.10E-186	0.489	0.704	0.039	1.95E-182	6
CENPM	4.06E-179	0.3659	0.691	0.038	7.24E-175	6

LMNB1	1.20E-171	0.40673	0.815	0.058	2.13E-167	6
PRR11	2.51E-165	0.43943	0.617	0.033	4.46E-161	6
AURKA	5.63E-158	0.7003	0.63	0.037	1.00E-153	6
CCNB1	6.81E-153	1.34701	0.877	0.083	1.21E-148	6
ZWINT	2.41E-148	0.43848	0.728	0.055	4.28E-144	6
AURKB	1.91E-145	0.29977	0.58	0.033	3.40E-141	6
CENPK	1.64E-138	0.4651	0.802	0.075	2.92E-134	6
FOXM1	1.03E-136	0.39423	0.716	0.057	1.83E-132	6
CDCA2	1.26E-135	0.28923	0.556	0.032	2.24E-131	6
TPX2	9.45E-131	1.23723	0.926	0.115	1.68E-126	6
TK1	2.83E-124	0.90796	0.963	0.135	5.03E-120	6
FANCI	3.43E-121	0.26428	0.58	0.041	6.11E-117	6
ASPM	4.02E-120	1.05142	0.815	0.091	7.17E-116	6
ARHGAP	4.59E-118	0.45282	0.704	0.064	8.18E-114	6
GINS2	1.67E-117	0.41528	0.642	0.053	2.97E-113	6
CIP2A	2.60E-108	0.3717	0.728	0.076	4.63E-104	6
MAD2L1	6.38E-106	0.83954	0.889	0.136	1.14E-101	6
CCNB2	5.17E-105	0.81971	0.852	0.116	9.20E-101	6
RACGAP	2.33E-102	0.35184	0.642	0.06	4.14E-98	6
KIF23	4.92E-101	0.57312	0.679	0.073	8.76E-97	6
CKAP2L	1.20E-100	0.40579	0.63	0.059	2.14E-96	6
UHRF1	3.30E-92	0.34999	0.691	0.081	5.88E-88	6
ORC6	4.69E-90	0.40898	0.667	0.078	8.34E-86	6
CENPW	2.45E-88	0.80258	0.975	0.214	4.36E-84	6
KIF20B	2.29E-85	0.82633	0.827	0.14	4.08E-81	6
MELK	2.33E-82	0.25044	0.543	0.053	4.14E-78	6
SGO2	2.35E-82	0.65188	0.704	0.098	4.18E-78	6
DIAPH3	1.15E-79	0.50039	0.79	0.129	2.04E-75	6
CDK1	2.50E-77	0.58591	0.519	0.052	4.46E-73	6
ECT2	2.64E-67	0.47739	0.765	0.142	4.70E-63	6
MCM5	3.63E-65	0.27245	0.531	0.064	6.46E-61	6
CENPH	6.98E-65	0.25454	0.716	0.115	1.24E-60	6
PHF19	1.44E-64	0.77163	0.926	0.27	2.57E-60	6
TYMS	3.44E-57	0.92693	0.988	0.412	6.12E-53	6
FEN1	1.21E-56	0.33724	0.679	0.125	2.16E-52	6
UBE2T	2.82E-53	0.62808	0.84	0.234	5.02E-49	6
PCLAF	3.95E-53	1.00001	0.975	0.426	7.03E-49	6
CENPN	6.63E-53	0.36958	0.84	0.204	1.18E-48	6
H2AFZ	1.06E-52	1.44892	1	0.982	1.89E-48	6
EZH2	3.74E-51	0.27148	0.667	0.125	6.66E-47	6
TUBA1B	6.94E-51	1.69608	1	0.965	1.24E-46	6
MCM7	1.32E-50	0.48136	0.815	0.222	2.35E-46	6
LMNB2	2.51E-50	0.4529	0.864	0.255	4.46E-46	6
HMGB1	3.30E-49	1.16076	1	0.986	5.87E-45	6
STMN1	4.57E-49	1.31644	1	0.86	8.14E-45	6
TUBB	6.01E-48	0.93775	1	0.985	1.07E-43	6
DEK	1.07E-47	0.9198	1	0.948	1.90E-43	6
MIS18A	1.14E-47	0.31996	0.741	0.178	2.03E-43	6
TUBB4B	1.38E-47	1.30935	0.988	0.844	2.45E-43	6
PTTG1	2.54E-47	1.47286	0.988	0.479	4.52E-43	6
HELLS	2.54E-47	0.44178	0.667	0.14	4.53E-43	6
VRK1	3.05E-47	0.30953	0.691	0.153	5.43E-43	6
CKS1B	3.06E-47	1.02426	0.951	0.485	5.44E-43	6
DTYMK	6.70E-47	0.7684	0.975	0.563	1.19E-42	6
SMC2	7.89E-46	0.60917	0.938	0.39	1.41E-41	6
NUCKS1	8.05E-45	0.87921	1	0.982	1.43E-40	6

UBE2S	3.60E-44	1.29958	1	0.846	6.41E-40	6
RAN	2.05E-43	0.70918	1	0.983	3.65E-39	6
CKAP2	2.41E-43	0.78118	0.753	0.226	4.29E-39	6
H2AFX	4.91E-43	0.66966	0.926	0.404	8.74E-39	6
HMGB2	6.08E-43	1.46175	0.951	0.61	1.08E-38	6
HNRNPA	1.22E-42	0.74633	1	0.977	2.17E-38	6
TUBA1C	1.29E-42	1.12291	0.988	0.943	2.30E-38	6
MCM4	1.68E-42	0.27267	0.556	0.107	2.99E-38	6
CKS2	5.16E-42	1.3002	0.889	0.399	9.20E-38	6
HMGB3	1.18E-41	0.72216	0.938	0.461	2.10E-37	6
KNSTRN	1.41E-41	0.41038	0.654	0.155	2.50E-37	6
RPA3	4.30E-41	0.58531	0.975	0.623	7.66E-37	6
PTMA	4.37E-41	0.68066	1	0.993	7.77E-37	6
HMGN2	6.91E-41	1.05535	1	0.977	1.23E-36	6
JPT1	9.01E-41	0.91248	0.988	0.902	1.60E-36	6
RANBP1	8.37E-40	0.75203	0.988	0.936	1.49E-35	6
RRM1	1.10E-39	0.45443	0.852	0.332	1.96E-35	6
NCAPD2	5.85E-39	0.43774	0.815	0.28	1.04E-34	6
KPNA2	1.44E-38	1.04182	0.951	0.564	2.56E-34	6
LSM5	2.69E-38	0.61662	1	0.884	4.79E-34	6
ANP32E	5.84E-38	0.73404	0.988	0.66	1.04E-33	6
DDX39A	1.71E-37	0.43661	0.889	0.351	3.05E-33	6
SMC4	1.05E-36	1.00264	0.975	0.593	1.86E-32	6
CAV1	2.60E-36	1.06244	1	0.935	4.62E-32	6
ATAD2	1.17E-35	0.34195	0.605	0.152	2.08E-31	6
RPL39L	1.68E-35	0.33894	0.889	0.358	2.98E-31	6
CFL1	3.74E-35	0.51159	1	0.991	6.65E-31	6
ENO1	1.07E-34	0.63326	1	0.979	1.90E-30	6
NASP	1.27E-34	0.59055	0.975	0.717	2.27E-30	6
LSM3	1.74E-34	0.55254	0.988	0.924	3.10E-30	6
H2AFV	2.76E-34	0.62626	1	0.939	4.92E-30	6
TUBA1A	3.58E-34	0.83614	1	0.974	6.38E-30	6
HSP90A/	5.43E-34	0.68309	1	0.986	9.67E-30	6
NSD2	1.01E-33	0.36513	0.765	0.269	1.79E-29	6
SMS	1.55E-33	0.60883	1	0.902	2.76E-29	6
ACTB	6.37E-33	0.53199	1	0.998	1.13E-28	6
PRKDC	2.44E-32	0.51257	0.988	0.919	4.35E-28	6
LSM4	2.93E-32	0.50311	1	0.884	5.21E-28	6
DUT	3.38E-32	0.56772	0.963	0.901	6.01E-28	6
DBF4	3.97E-32	0.42217	0.704	0.237	7.07E-28	6
ILF2	4.31E-32	0.49188	0.988	0.938	7.68E-28	6
NUDT1	6.30E-32	0.45831	0.951	0.549	1.12E-27	6
PTGES3	8.92E-32	0.50487	1	0.97	1.59E-27	6
ANP32B	9.15E-32	0.48408	1	0.977	1.63E-27	6
HNRNPD	1.16E-31	0.53978	0.988	0.913	2.07E-27	6
LRR1	2.07E-31	0.26314	0.679	0.2	3.69E-27	6
CCDC34	2.90E-31	0.31658	0.79	0.277	5.16E-27	6
RANGAP	3.17E-31	0.34337	0.778	0.286	5.64E-27	6
SFPQ	3.33E-31	0.62291	0.975	0.925	5.93E-27	6
CBX5	4.12E-31	0.53784	0.975	0.773	7.33E-27	6
SNRPG	5.77E-31	0.44165	1	0.971	1.03E-26	6
SRSF7	7.30E-31	0.65452	0.988	0.878	1.30E-26	6
CALM1	7.80E-31	0.49385	1	0.979	1.39E-26	6
MZT1	8.58E-31	0.51832	0.951	0.57	1.53E-26	6
SNRPB	1.12E-30	0.54588	0.975	0.932	1.99E-26	6
EXOSC8	1.26E-30	0.44401	0.938	0.578	2.25E-26	6

SET	1.37E-30	0.46691	1	0.989	2.44E-26	6
LDHA	1.49E-30	0.57716	1	0.97	2.66E-26	6
DNAJC9	2.85E-30	0.3952	0.778	0.318	5.07E-26	6
HSPD1	3.30E-30	0.54539	1	0.963	5.87E-26	6
ITGB3BP	3.62E-30	0.26368	0.704	0.222	6.44E-26	6
TPM3	4.13E-30	0.60515	1	0.961	7.36E-26	6
KPNB1	8.95E-30	0.51843	0.988	0.957	1.59E-25	6
TPI1	1.28E-29	0.49382	1	0.97	2.27E-25	6
CSE1L	1.95E-29	0.30065	0.852	0.357	3.47E-25	6
C12orf75	2.17E-29	0.66131	1	0.968	3.87E-25	6
SNRPD1	4.31E-29	0.5364	0.975	0.943	7.68E-25	6
GMNN	4.83E-29	0.37684	0.741	0.293	8.60E-25	6
HNRNPA	6.06E-29	0.56602	1	0.935	1.08E-24	6
DHFR	7.49E-29	0.32502	0.778	0.308	1.33E-24	6
TMPO	1.86E-28	0.63071	0.901	0.589	3.32E-24	6
DNMT1	4.52E-28	0.52891	0.889	0.566	8.05E-24	6
SNRPA1	4.73E-28	0.44422	0.938	0.596	8.42E-24	6
TUBB6	7.44E-28	0.63896	0.975	0.897	1.32E-23	6
EIF5A	1.84E-27	0.52031	1	0.976	3.28E-23	6
YWHAZ	2.09E-27	0.44182	1	0.972	3.72E-23	6
ARL6IP1	6.71E-27	0.81471	0.926	0.623	1.19E-22	6
CCT5	8.61E-27	0.49428	0.975	0.922	1.53E-22	6
ODC1	1.07E-26	0.59817	0.988	0.829	1.91E-22	6
HNRNPR	1.14E-26	0.45526	0.988	0.927	2.03E-22	6
EIF1AX	2.37E-26	0.46277	0.988	0.961	4.22E-22	6
PFN1	3.46E-26	0.42027	1	0.992	6.17E-22	6
PSMD2	6.86E-26	0.48291	0.988	0.91	1.22E-21	6
MCM3	1.67E-25	0.29976	0.58	0.173	2.98E-21	6
BARD1	2.01E-25	0.25771	0.556	0.154	3.58E-21	6
PRELID1	2.01E-25	0.38819	1	0.979	3.58E-21	6
BCL2L12	2.61E-25	0.27461	0.704	0.267	4.65E-21	6
SRSF2	2.82E-25	0.50974	0.963	0.914	5.02E-21	6
CBX1	3.29E-25	0.48067	0.975	0.852	5.85E-21	6
PA2G4	3.71E-25	0.46532	0.988	0.92	6.61E-21	6
LMNA	3.74E-25	0.45981	1	0.972	6.67E-21	6
SUN2	5.40E-25	0.27117	0.728	0.264	9.61E-21	6
HMGN1	6.00E-25	0.40259	1	0.986	1.07E-20	6
PTMS	6.19E-25	0.48155	1	0.987	1.10E-20	6
COTL1	7.76E-25	0.52323	1	0.971	1.38E-20	6
HNRNPA	8.08E-25	0.49422	1	0.948	1.44E-20	6
CLIC1	9.06E-25	0.4103	1	0.98	1.61E-20	6
YBX1	1.32E-24	0.29845	1	0.993	2.34E-20	6
SRSF3	2.09E-24	0.55454	0.988	0.951	3.72E-20	6
LSM2	3.13E-24	0.4087	0.988	0.808	5.57E-20	6
FABP5	3.57E-24	0.60989	0.963	0.826	6.36E-20	6
TMSB10	3.72E-24	0.29066	1	1	6.63E-20	6
HNRNPH	3.90E-24	0.46406	0.975	0.855	6.94E-20	6
GSPT1	5.09E-24	0.41783	1	0.886	9.06E-20	6
HNRNPU	7.07E-24	0.50173	1	0.957	1.26E-19	6
SNRPE	7.93E-24	0.3889	0.988	0.972	1.41E-19	6
EIF4G2	2.67E-23	0.3613	1	0.979	4.75E-19	6
HDGF	2.68E-23	0.41755	1	0.91	4.76E-19	6
MAGOHE	2.77E-23	0.37777	0.963	0.722	4.94E-19	6
PSMA7	2.81E-23	0.35701	1	0.981	5.00E-19	6
SERBP1	3.39E-23	0.37386	1	0.981	6.04E-19	6
SRP9	3.82E-23	0.41359	1	0.959	6.79E-19	6

XRCC5	5.28E-23	0.43348	0.988	0.96	9.41E-19	6
TAGLN2	8.21E-23	0.43193	1	0.948	1.46E-18	6
HNRNPM	9.05E-23	0.46294	1	0.907	1.61E-18	6
CMSS1	1.05E-22	0.36086	0.914	0.534	1.86E-18	6
CENPX	1.09E-22	0.41563	0.938	0.681	1.94E-18	6
TPRKB	1.73E-22	0.41275	0.926	0.7	3.08E-18	6
YWHAH	1.91E-22	0.47674	0.988	0.87	3.40E-18	6
MAP1B	2.16E-22	0.44463	1	0.982	3.84E-18	6
BUB3	2.36E-22	0.46045	0.938	0.728	4.21E-18	6
HNRNPD	2.62E-22	0.40739	1	0.959	4.67E-18	6
NCL	3.04E-22	0.48574	1	0.975	5.41E-18	6
ARPC2	3.24E-22	0.33203	1	0.99	5.77E-18	6
SEPHS1	3.30E-22	0.31067	0.889	0.504	5.88E-18	6
TPM4	3.99E-22	0.41247	1	0.992	7.11E-18	6
SKA2	4.55E-22	0.43178	0.938	0.718	8.11E-18	6
HIST1H4	4.76E-22	1.12169	0.975	0.915	8.47E-18	6
ALYREF	7.43E-22	0.3639	0.938	0.645	1.32E-17	6
CHCHD2	8.03E-22	0.26586	1	0.99	1.43E-17	6
RBMX	9.13E-22	0.38257	0.988	0.838	1.63E-17	6
RHEB	2.41E-21	0.35691	1	0.954	4.29E-17	6
FBXO5	4.36E-21	0.26829	0.543	0.177	7.76E-17	6
DNAJA1	5.44E-21	0.39064	1	0.941	9.69E-17	6
COX8A	5.90E-21	0.31944	1	0.984	1.05E-16	6
LBR	7.06E-21	0.34732	0.815	0.41	1.26E-16	6
ID1	8.33E-21	0.45757	0.79	0.347	1.48E-16	6
PCNA	9.04E-21	0.45537	0.877	0.566	1.61E-16	6
ABCE1	1.22E-20	0.34715	0.975	0.747	2.17E-16	6
SMC1A	1.44E-20	0.39947	0.864	0.545	2.56E-16	6
PSIP1	1.52E-20	0.37892	0.951	0.787	2.71E-16	6
CENPV	1.66E-20	0.43276	0.852	0.522	2.95E-16	6
KHDRBS	2.14E-20	0.36927	0.988	0.937	3.80E-16	6
POLD2	2.48E-20	0.31249	0.951	0.755	4.41E-16	6
SNRPF	7.91E-20	0.36026	0.988	0.966	1.41E-15	6
HSP90AE	1.06E-19	0.41981	1	0.987	1.89E-15	6
HSPE1	1.25E-19	0.41191	0.988	0.969	2.23E-15	6
PHB	1.30E-19	0.32653	1	0.927	2.31E-15	6
SLC25A5	1.96E-19	0.34317	1	0.958	3.48E-15	6
CACYBP	3.85E-19	0.4181	0.938	0.787	6.85E-15	6
PGP	3.92E-19	0.3287	0.901	0.684	6.99E-15	6
SNRPD3	5.55E-19	0.2972	1	0.954	9.88E-15	6
NAP1L4	6.62E-19	0.4147	0.963	0.806	1.18E-14	6
PPP1R14	7.66E-19	0.34698	1	0.986	1.36E-14	6
HSPA8	9.02E-19	0.50961	1	0.976	1.61E-14	6
RAD21	9.12E-19	0.47974	0.975	0.884	1.62E-14	6
EIF2S1	1.11E-18	0.30164	0.975	0.807	1.98E-14	6
SMC3	1.15E-18	0.35953	0.938	0.804	2.05E-14	6
XRCC6	1.28E-18	0.33698	0.988	0.911	2.28E-14	6
GPN3	1.60E-18	0.28632	0.827	0.475	2.84E-14	6
YWHAE	2.08E-18	0.30971	1	0.986	3.69E-14	6
SYNCRIF	2.08E-18	0.36211	0.988	0.918	3.70E-14	6
TMEM10	2.29E-18	0.26084	0.914	0.565	4.08E-14	6
FOSL1	2.32E-18	0.29298	0.654	0.274	4.13E-14	6
SSRP1	5.49E-18	0.425	0.926	0.729	9.78E-14	6
BANF1	6.25E-18	0.30334	0.988	0.933	1.11E-13	6
AURKAIF	7.94E-18	0.29082	1	0.965	1.41E-13	6
PSMA4	8.63E-18	0.29066	1	0.955	1.54E-13	6

PFKP	9.27E-18	0.30135	0.926	0.584	1.65E-13	6
PSMC3	9.58E-18	0.28653	0.988	0.902	1.70E-13	6
PCMT1	1.02E-17	0.29501	0.963	0.851	1.81E-13	6
G3BP1	1.07E-17	0.3112	0.975	0.867	1.91E-13	6
HNRNPC	1.10E-17	0.34248	1	0.973	1.96E-13	6
PPM1G	1.53E-17	0.34313	0.963	0.896	2.72E-13	6
BOLA3	1.85E-17	0.31771	0.938	0.779	3.29E-13	6
KIF22	2.97E-17	0.26906	0.679	0.312	5.28E-13	6
STIP1	3.09E-17	0.30697	0.951	0.675	5.50E-13	6
HNRNPK	3.22E-17	0.33498	0.975	0.976	5.73E-13	6
TCP1	3.33E-17	0.31299	0.963	0.887	5.93E-13	6
CDC25B	3.89E-17	0.3011	0.642	0.283	6.93E-13	6
CCT2	3.93E-17	0.33734	0.988	0.939	7.00E-13	6
DYNLL1	3.94E-17	0.30737	1	0.989	7.01E-13	6
PMAIP1	3.98E-17	0.30027	0.728	0.357	7.10E-13	6
MSN	4.29E-17	0.34185	0.975	0.908	7.64E-13	6
C1QBP	4.68E-17	0.3423	1	0.961	8.33E-13	6
ERH	5.65E-17	0.27745	1	0.97	1.01E-12	6
ACTR2	8.22E-17	0.33785	0.988	0.938	1.46E-12	6
BZW1	8.75E-17	0.3386	0.975	0.952	1.56E-12	6
TOMM40	9.05E-17	0.33154	0.864	0.633	1.61E-12	6
EIF5	9.41E-17	0.31845	1	0.976	1.68E-12	6
MRTO4	9.42E-17	0.33498	0.901	0.639	1.68E-12	6
CALM3	1.01E-16	0.3456	1	0.9	1.81E-12	6
PRDX1	1.09E-16	0.28608	0.988	0.979	1.95E-12	6
TM4SF1	1.10E-16	0.43439	0.728	0.38	1.96E-12	6
HNRNPH	1.11E-16	0.32809	0.975	0.863	1.98E-12	6
HDAC2	1.25E-16	0.30741	0.975	0.809	2.23E-12	6
ANP32A	1.43E-16	0.33236	0.951	0.78	2.55E-12	6
YWHAQ	1.72E-16	0.34294	0.975	0.971	3.06E-12	6
COX20	1.84E-16	0.36437	1	0.909	3.28E-12	6
XPO1	1.91E-16	0.33103	0.889	0.745	3.39E-12	6
TUBB2A	1.95E-16	0.36802	0.938	0.702	3.47E-12	6
ACTN4	2.47E-16	0.38142	0.963	0.904	4.40E-12	6
EBNA1BI	2.50E-16	0.34553	0.901	0.698	4.45E-12	6
7-Sep	2.59E-16	0.30382	1	0.975	4.61E-12	6
SRRM1	2.81E-16	0.30113	0.988	0.922	5.00E-12	6
SPDL1	2.88E-16	0.38396	0.778	0.451	5.13E-12	6
U2SURP	3.24E-16	0.31399	0.975	0.801	5.77E-12	6
PGK1	5.33E-16	0.29327	0.975	0.937	9.49E-12	6
GLO1	6.72E-16	0.27139	0.988	0.901	1.20E-11	6
MIS18BP	7.42E-16	0.30541	0.877	0.593	1.32E-11	6
CCT6A	7.79E-16	0.31129	0.988	0.968	1.39E-11	6
PGAM1	9.79E-16	0.30304	0.988	0.967	1.74E-11	6
USP1	1.05E-15	0.29897	0.778	0.476	1.86E-11	6
USP14	1.12E-15	0.29079	0.963	0.844	1.99E-11	6
DHX9	1.13E-15	0.27902	0.951	0.645	2.01E-11	6
CKAP5	1.14E-15	0.32026	0.765	0.449	2.02E-11	6
POP7	1.14E-15	0.28299	0.877	0.676	2.02E-11	6
PHGDH	1.15E-15	0.3287	0.975	0.958	2.05E-11	6
FUS	1.34E-15	0.33742	0.975	0.948	2.38E-11	6
RNF145	1.45E-15	0.36895	0.914	0.662	2.57E-11	6
ANXA1	1.50E-15	0.38907	1	0.978	2.67E-11	6
DKC1	1.96E-15	0.31038	0.914	0.67	3.49E-11	6
HNRNPA	2.17E-15	0.31994	1	0.989	3.86E-11	6
NME1	2.31E-15	0.31935	1	0.956	4.11E-11	6

TRA2B	2.45E-15	0.32834	0.951	0.77	4.36E-11	6
RBM8A	2.45E-15	0.33978	0.988	0.936	4.36E-11	6
LRRC59	2.63E-15	0.31188	0.975	0.909	4.69E-11	6
SAE1	2.85E-15	0.2724	0.827	0.573	5.08E-11	6
PAICS	3.01E-15	0.32415	0.852	0.689	5.37E-11	6
HSPH1	3.41E-15	0.32649	0.926	0.742	6.07E-11	6
MRPL51	3.62E-15	0.28289	1	0.979	6.45E-11	6
FUBP1	3.66E-15	0.26638	0.84	0.539	6.51E-11	6
MYH9	3.82E-15	0.3422	0.988	0.964	6.81E-11	6
DHCR24	3.92E-15	0.30462	0.901	0.673	6.98E-11	6
PSMD14	4.16E-15	0.32442	0.963	0.848	7.40E-11	6
COMMD4	4.43E-15	0.28028	0.988	0.835	7.89E-11	6
PDAP1	4.55E-15	0.29713	1	0.95	8.09E-11	6
SLIRP	6.11E-15	0.28851	0.975	0.962	1.09E-10	6
SF3B2	7.21E-15	0.29607	1	0.874	1.28E-10	6
CYCS	7.47E-15	0.3836	0.975	0.907	1.33E-10	6
CKLF	1.42E-14	0.30367	0.889	0.793	2.52E-10	6
ATP5F1E	1.49E-14	0.27199	0.963	0.963	2.65E-10	6
CAVIN1	1.73E-14	0.33867	0.988	0.978	3.07E-10	6
DDX21	1.79E-14	0.36931	0.975	0.872	3.19E-10	6
YWHAB	1.83E-14	0.2597	1	0.981	3.25E-10	6
FLNA	1.87E-14	0.3154	1	0.974	3.34E-10	6
RDX	1.89E-14	0.31043	0.963	0.922	3.36E-10	6
SUPT16H	3.31E-14	0.30487	0.951	0.831	5.89E-10	6
MTDH	3.90E-14	0.26564	0.988	0.971	6.95E-10	6
THRAP3	4.27E-14	0.30012	0.963	0.917	7.61E-10	6
HAT1	4.39E-14	0.26407	0.728	0.497	7.82E-10	6
ARPC5L	5.01E-14	0.31768	0.963	0.834	8.92E-10	6
GNAI2	6.34E-14	0.26382	1	0.976	1.13E-09	6
UBE2N	6.35E-14	0.26021	0.926	0.843	1.13E-09	6
DNAJC8	6.47E-14	0.31093	1	0.954	1.15E-09	6
CNBP	1.02E-13	0.2911	0.988	0.934	1.82E-09	6
SNRPC	1.40E-13	0.25138	1	0.897	2.48E-09	6
TMEM16[illegible]	1.42E-13	0.27195	0.975	0.793	2.52E-09	6
ILF3	1.42E-13	0.29582	0.938	0.786	2.53E-09	6
KIF5B	1.44E-13	0.38196	0.988	0.948	2.57E-09	6
HRAS	1.51E-13	0.26152	0.988	0.858	2.69E-09	6
GNB1	1.62E-13	0.28267	0.988	0.941	2.88E-09	6
CAV2	1.65E-13	0.32948	0.926	0.718	2.93E-09	6
ACTR3	1.69E-13	0.30335	0.963	0.944	3.01E-09	6
CAP1	2.00E-13	0.29652	0.988	0.965	3.56E-09	6
ACTN1	2.15E-13	0.30259	0.988	0.967	3.83E-09	6
MYL6	2.85E-13	0.27392	1	0.993	5.07E-09	6
WDR1	2.86E-13	0.2623	0.975	0.918	5.09E-09	6
NOP56	3.19E-13	0.30621	0.951	0.793	5.68E-09	6
MAP3K2[illegible]	4.29E-13	0.25333	0.901	0.801	7.63E-09	6
FOXD1	5.85E-13	0.33053	0.827	0.566	1.04E-08	6
ARHGDIA	5.92E-13	0.28592	0.975	0.922	1.05E-08	6
ID3	7.35E-13	0.51207	0.926	0.737	1.31E-08	6
NOP58	7.41E-13	0.27879	0.815	0.578	1.32E-08	6
PRMT1	9.08E-13	0.26443	0.975	0.941	1.62E-08	6
ANAPC1[illegible]	9.09E-13	0.25968	1	0.974	1.62E-08	6
MRPL52	1.07E-12	0.28079	1	0.962	1.91E-08	6
H3F3B	1.13E-12	0.31101	1	0.988	2.02E-08	6
VDAC3	1.24E-12	0.30277	0.963	0.86	2.22E-08	6
EBP	1.26E-12	0.28236	0.889	0.682	2.25E-08	6

RTN4	1.28E-12	0.25721	0.988	0.984	2.28E-08	6
PSMD11	1.33E-12	0.27563	0.975	0.855	2.37E-08	6
MARCKS	1.53E-12	0.28574	1	0.99	2.73E-08	6
VBP1	1.88E-12	0.27651	0.938	0.772	3.35E-08	6
KCNMA1	2.32E-12	0.41767	0.938	0.748	4.12E-08	6
ITGB1BP	2.35E-12	0.25865	0.988	0.925	4.19E-08	6
EIF3A	2.36E-12	0.27595	1	0.977	4.21E-08	6
CORO1C	2.77E-12	0.26958	0.901	0.697	4.93E-08	6
TPR	3.02E-12	0.26394	0.963	0.921	5.37E-08	6
DAZAP1	3.55E-12	0.26841	0.914	0.736	6.32E-08	6
CNIH4	3.56E-12	0.26516	0.975	0.904	6.34E-08	6
LRRFIP1	4.21E-12	0.29846	0.975	0.817	7.49E-08	6
MT2A	4.67E-12	0.40543	1	0.997	8.31E-08	6
HMGN3	5.78E-12	0.25449	0.963	0.777	1.03E-07	6
ADRM1	5.98E-12	0.25575	0.988	0.872	1.07E-07	6
NHP2	6.32E-12	0.29202	1	0.946	1.13E-07	6
CBX3	6.89E-12	0.27325	0.975	0.965	1.23E-07	6
ASPH	7.08E-12	0.27768	1	0.949	1.26E-07	6
PLP2	1.11E-11	0.28007	0.988	0.899	1.97E-07	6
ACAT2	1.25E-11	0.3317	0.988	0.865	2.23E-07	6
PITX1	1.75E-11	0.30826	0.975	0.87	3.11E-07	6
NOL7	1.84E-11	0.25543	0.975	0.947	3.28E-07	6
FSCN1	2.11E-11	0.25364	0.988	0.92	3.76E-07	6
SON	2.19E-11	0.27692	0.988	0.964	3.90E-07	6
KMT5A	2.27E-11	0.32307	0.802	0.621	4.05E-07	6
TIMM10	2.51E-11	0.27097	0.914	0.849	4.47E-07	6
DRAP1	2.52E-11	0.2859	1	0.968	4.48E-07	6
NQO1	2.59E-11	0.34585	0.938	0.81	4.61E-07	6
RFLNB	2.84E-11	0.34113	0.84	0.697	5.06E-07	6
PSMD1	3.59E-11	0.25782	0.988	0.888	6.40E-07	6
ALCAM	4.37E-11	0.28853	0.889	0.605	7.77E-07	6
CMC2	4.93E-11	0.25183	0.864	0.698	8.78E-07	6
NUDC	6.18E-11	0.27809	0.951	0.889	1.10E-06	6
DDX46	7.94E-11	0.25261	1	0.893	1.41E-06	6
EZR	1.18E-10	0.2765	0.802	0.574	2.11E-06	6
H1FX	2.13E-10	0.31839	0.877	0.697	3.79E-06	6
RUVBL1	2.20E-10	0.25051	0.864	0.692	3.92E-06	6
PHLDA1	8.35E-10	0.38211	0.951	0.82	1.49E-05	6
EIF4EBP	9.24E-10	0.25789	1	0.976	1.64E-05	6
JUN	9.57E-10	0.31134	0.864	0.634	1.70E-05	6
ACTG2	9.69E-10	0.39112	0.877	0.64	1.73E-05	6
AXL	1.33E-09	0.25654	0.914	0.758	2.37E-05	6
CCND1	2.04E-09	0.43096	0.988	0.93	3.63E-05	6
LMO7	4.72E-09	0.25594	0.914	0.746	8.41E-05	6
PSME2	4.02E-08	0.25255	0.963	0.843	0.000715511	6
HMGA1	7.08E-08	0.26333	0.988	0.966	0.001260547	6
OGFRL1	1.11E-07	0.2676	0.765	0.539	0.001973351	6
SLBP	3.91E-07	0.25238	0.864	0.725	0.006962911	6
THBS1	4.42E-07	0.34134	0.963	0.823	0.007863857	6
DCBLD2	5.14E-07	0.27851	0.951	0.854	0.009144384	6

Supplementary Table 4. Top 10 of DEGs among different clusters

gene	p_val	avg_logF	pct.1	pct.2	p_val_adj	cluster
SPON2	3.69E-189	0.39714	0.996	0.939	6.57E-185	0
CTSK	9.05E-142	0.37391	0.768	0.543	1.61E-137	0
MDK	1.48E-137	0.34762	0.995	0.948	2.63E-133	0
RBP1	7.67E-119	0.33416	0.993	0.941	1.37E-114	0
POSTN	5.20E-114	0.45306	0.734	0.494	9.27E-110	0
CXCL6	1.00E-105	0.47737	0.688	0.487	1.79E-101	0
TMEM15	2.81E-81	0.32866	0.875	0.792	5.00E-77	0
CXCL1	1.67E-62	0.64776	0.592	0.433	2.98E-58	0
CHI3L1	5.40E-35	0.44729	0.628	0.509	9.61E-31	0
CCL2	7.94E-34	0.33313	0.926	0.881	1.41E-29	0
RHOBTB	0	0.51053	1	0.966	0	1
TUBA1A	1.79E-297	0.51083	1	0.965	3.18E-293	1
GLRX	1.05E-276	0.5234	0.998	0.935	1.86E-272	1
TUBA1B	5.29E-270	0.56529	0.997	0.955	9.42E-266	1
THBS1	1.42E-251	0.5453	0.964	0.777	2.53E-247	1
CTGF	1.92E-234	0.69995	0.959	0.747	3.41E-230	1
CAV1	4.27E-211	0.60288	0.986	0.919	7.60E-207	1
CYR61	1.71E-205	0.53575	0.972	0.817	3.04E-201	1
DKK1	3.04E-204	0.59021	0.912	0.665	5.41E-200	1
PTX3	3.87E-198	0.68276	0.998	0.955	6.88E-194	1
LUM	0	0.6424	1	0.978	0	2
DCN	7.47E-234	0.4976	1	0.98	1.33E-229	2
CTSL	1.46E-230	0.43191	0.993	0.935	2.59E-226	2
TFPI2	1.92E-212	0.74485	0.951	0.719	3.42E-208	2
HTRA1	1.07E-207	0.39763	0.956	0.755	1.91E-203	2
SERPINF	7.11E-189	0.38619	0.977	0.84	1.27E-184	2
VCAN	2.19E-148	0.34651	1	0.979	3.90E-144	2
GNG11	8.13E-138	0.3443	0.988	0.935	1.45E-133	2
CLU	5.70E-85	0.35298	0.99	0.956	1.01E-80	2
CHI3L1	1.95E-68	0.62276	0.684	0.498	3.48E-64	2
SERPINE	0	1.18668	1	0.974	0	3
PRSS23	4.66E-277	0.778	0.988	0.695	8.30E-273	3
TAGLN	1.44E-255	0.82778	1	0.989	2.57E-251	3
CCDC80	9.84E-235	0.77106	1	0.982	1.75E-230	3
TPM1	1.83E-227	0.77821	1	0.992	3.25E-223	3
SERPINE	6.02E-226	0.84714	0.998	0.875	1.07E-221	3
SCRG1	6.18E-217	0.69711	0.904	0.476	1.10E-212	3
ACTA2	9.63E-213	1.22859	0.992	0.919	1.71E-208	3
XRCC4	1.67E-186	0.73055	0.784	0.367	2.97E-182	3
CTGF	4.56E-94	0.68939	0.959	0.78	8.11E-90	3
SPARC	1.34E-164	0.51998	1	0.988	2.39E-160	4
LOX	3.80E-160	0.62753	1	0.961	6.77E-156	4
IGFBP7	1.01E-151	0.63936	1	0.964	1.79E-147	4
TIMP1	1.63E-126	0.6321	1	0.986	2.91E-122	4
IFI6	4.48E-120	0.66027	0.957	0.766	7.99E-116	4
CDKN1A	2.83E-90	0.45249	1	0.919	5.03E-86	4
SPON2	8.69E-76	0.41228	0.994	0.949	1.55E-71	4
NDUFA4I	1.08E-70	0.41027	0.761	0.428	1.93E-66	4
TIMP3	4.80E-65	0.57026	0.991	0.863	8.55E-61	4
SERPINE	1.95E-54	0.49267	1	0.975	3.46E-50	4
PABPC1	7.53E-45	0.39394	0.826	1	1.34E-40	5
LRRC75/	2.42E-39	1.10151	0.653	0.724	4.32E-35	5
SET	6.56E-21	0.35391	0.815	1	1.17E-16	5

PHKG1	7.32E-17	0.44925	0.399	0.308	1.30E-12	5
CLDN11	7.38E-10	0.53719	0.709	0.991	1.31E-05	5
TOP2A	0	1.36413	0.889	0.033	0	6
CENPF	3.54E-198	1.86088	0.914	0.068	6.30E-194	6
CCNB1	6.81E-153	1.34701	0.877	0.083	1.21E-148	6
H2AFZ	1.06E-52	1.44892	1	0.982	1.89E-48	6
TUBA1B	6.94E-51	1.69608	1	0.965	1.24E-46	6
STMN1	4.57E-49	1.31644	1	0.86	8.14E-45	6
TUBB4B	1.38E-47	1.30935	0.988	0.844	2.45E-43	6
PTTG1	2.54E-47	1.47286	0.988	0.479	4.52E-43	6
HMGB2	6.08E-43	1.46175	0.951	0.61	1.08E-38	6
CKS2	5.16E-42	1.3002	0.889	0.399	9.20E-38	6

Supplementary Table 5. DEGs between CD317+ vs CD317- MSCs

Table S5.1. Differential expressed genes between CD317+ vs CD317- MSCs

Gene ID	Gene Symbol	Average FPKM		log2	Qvalue
		CD317-	CD317+		
100529097	'RPL36A-HNRNI	9.35	3.155	1.66861419	0.037241149
55586	'MIOX'	8.395	3.65	1.629147804	0.041274447
102723728	'LOC102723728	170.72	85.475	1.090133799	0.001348594
642778	'NPIPA3'	48.465	26.76	0.969775441	0.01103209
101059938	'NPIPA7'	351.945	201.89	0.891305575	0.02200956
54985	'HCFC1R1'	236.47	138.37	0.86791218	0.004429752
9997	'SCO2'	132.185	78.985	0.83743581	0.006794962
9314	'KLF4'	53.46	32.015	0.8332664	0.036021511
9284	'NPIPA1'	324.46	200.905	0.792262934	0.034442699
6206	'RPS12'	1880.72	1296.27	0.652722777	0.045903259
10952	'SEC61B'	747.015	516.35	0.63917866	0.02200956
6187	'RPS2'	7272.665	5089.41	0.614238474	0.042929565
6193	'RPS5'	1666.51	1173.535	0.607827894	0.02200956
6156	'RPL30'	3991.475	2885.52	0.581176619	0.021694092
5698	'PSMB9'	650.72	472.93	0.554383283	0.037590926
4627	'MYH9'	362.565	557.3	-0.535963246	0.037590926
6711	'SPTBN1'	63.995	98.34	-0.539966717	0.045216793
131566	'DCBLD2'	56.715	88.18	-0.574882505	0.037590926
23345	'SYNE1'	39.755	37.49	-0.584483479	0.045903259
57169	'ZNFX1'	49.985	79.78	-0.587621497	0.02691323
57724	'EPG5'	13.955	23.745	-0.589736791	0.029797792
81792	'ADAMTS12'	54.005	70.28	-0.59356931	0.034077747
154810	'AMOTL1'	17.61	27.885	-0.593603435	0.045216793
57493	'HEG1'	43.315	69.045	-0.599473887	0.037590926
84162	'KIAA1109'	6.87	10.855	-0.622465465	0.037590926
9510	'ADAMTS1'	22.615	37.425	-0.641279305	0.02691323
4638	'MYLK'	35.05	45.59	-0.646034837	0.02200956
64750	'SMURF2'	23.515	38.17	-0.651299974	0.023829962
84181	'CHD6'	10.43	15.035	-0.659778516	0.037241149
2195	'FAT1'	17.185	29.24	-0.687964518	0.004429752
23213	'SULF1'	311.685	507.875	-0.693366855	0.010328886
79987	'SVEP1'	4.535	7.915	-0.714252076	0.034077747
8204	'NRIP1'	19.47	33.14	-0.732581479	0.026697521
100462981	'MTRNR2L2'	4170.725	7588.81	-0.764168191	0.037590926
4035	'LRP1'	136.54	246.085	-0.767758476	0.005602611
57608	'JCAD'	15.89	28.985	-0.783479706	0.001765586
6240	'RRM1'	9.61	17.47	-0.797536536	0.046540057
1948	'EFNB2'	12.595	23.295	-0.804080163	0.037241149
9353	'SLIT2'	51.76	85.765	-0.82122567	1.63E-04
57685	'CACHD1'	4.805	9.005	-0.828938569	0.030753955
23220	'DTX4'	4.61	8.525	-0.833158846	0.043380079
9415	'FADS2'	72.385	136.82	-0.845478234	0.01784088
3097	'HIVEP2'	11.515	22.075	-0.860242945	0.003035686
23312	'DMXL2'	2.64	4.945	-0.926308811	0.030843263
3339	'HSPG2'	63.105	128.34	-0.949595715	0.02200956
3575	'IL7R'	19.035	38.84	-0.95445079	0.022407305
408050	'NOMO3'	22.19	45.75	-0.965708616	0.034442699
157506	'RDH10'	5.68	11.89	-0.975165507	0.038629074
80144	'FRAS1'	2.4	5.05	-0.994238538	0.003874214
1832	'DSP'	15.44	30.86	-1.01055356	1.63E-04
57211	'ADGRG6'	4.715	10.585	-1.101226279	0.001156743

7130	TNFAIP6'	233.43	523.22	-1.101568729	7.90E-07
5649	'RELN'	3.435	7.905	-1.101808375	0.037590926
1718	'DHCR24'	6.175	14.415	-1.135023294	5.67E-04
5426	'POLE'	1.825	4.62	-1.137720799	0.01516403
3949	'LDLR'	13.155	29.915	-1.148647746	8.30E-07
4171	'MCM2'	4.68	11.275	-1.175281939	0.005658208
3909	'LAMA3'	1.835	4.15	-1.178997987	0.02200956
6581	'SLC22A3'	17.15	42.03	-1.192311813	1.63E-04
4174	'MCM5'	6.89	16.91	-1.210615009	3.03E-04
7057	THBS1'	25.305	61.115	-1.23536187	0.022506984
684	CD317'	334.45	829.11	-1.23859675	1.52E-05
6241	RRM2'	3.05	7.635	-1.240473955	0.021073885
7052	TGM2'	107.89	276.23	-1.27053676	4.56E-08
6347	CCL2'	1343.36	3527.97	-1.27198546	6.10E-07
8507	ENC1'	24.33	59.94	-1.272232202	3.03E-04
1038	'CDR1'	12.43	32.385	-1.297389797	0.007282574
3557	IL1RN'	1.01	2.68	-1.308547685	3.90E-08
57575	'PCDH10'	5.23	16.055	-1.352355863	1.76E-06
7292	TNFSF4'	6.01	17.05	-1.418690797	0.010565241
5915	'RARB'	2.86	7.77	-1.479017315	0.006736355
100381270	'ZBED6'	2.525	7.93	-1.567000253	0.001933931
1311	COMP'	1.535	4.91	-1.588763533	0.037241149
171024	SYNPO2'	0.64	2.505	-1.73455151	0.021073885
27063	ANKRD1'	4.15	15.735	-1.839038149	1.63E-04
100293534	C4B_2'	1.72	6.815	-1.907624232	4.38E-07
195828	ZNF367'	0.255	1.735	-2.675109526	0.014008579

Supplementary Table 5. DEGs between CD317+ vs CD317- MSCs

Table S5.2. Expressed genes only in CD317+ MSCs

Gene ID	Gene Symbol	Average FPKM	
		CD317+	CD317-
402317	OR2A42'	2.39	0
10406	WFDC2'	0.745	0
128864	'C20orf144'	0.665	0
401427	'OR2A7'	0.66	0
283106	'CSNK2A3'	0.59	0
64073	'C19orf33'	0.51	0
8366	'H4C2'	0.48	0
5950	'RBP4'	0.425	0
8360	'H4C4'	0.425	0
345	'APOC3'	0.42	0
114108587	'ATF7-NPFF'	0.395	0
10850	'CCL27'	0.39	0
121504	'H4-16'	0.385	0
480	'ATP1A4'	0.355	0
1474	'CST6'	0.35	0
107987067	'LOC107987067'	0.325	0
1410	'CRYAB'	0.315	0
115677	'NOSTRIN'	0.31	0
283152	'CCDC153'	0.305	0
158511	'CSAG1'	0.295	0
102800317	'TPTEP2-CSNK1E'	0.29	0
653505	'PPIAL4A'	0.28	0
8342	'H2BC14'	0.28	0
6887	'TAL2'	0.275	0
219968	'OR5B21'	0.27	0
3447	'IFNA13'	0.27	0
79608	'RIC3'	0.265	0
8332	'H2AC16'	0.26	0
3018	'H2BC3'	0.255	0
100529241	'HSPE1-MOB4'	0.25	0
2695	'GIP'	0.25	0
3963	'LGALS7'	0.25	0
100130771	'EFCAB10'	0.24	0
885	'CCK'	0.235	0
100130827	'SBK3'	0.22	0
2571	'GAD1'	0.22	0
105373989	'LOC105373989'	0.215	0
112267886	'LOC112267886'	0.21	0
152816	'ODAPH'	0.21	0
3316	'HSPB2'	0.19	0
440955	'TMEM89'	0.185	0
3738	'KCNA3'	0.175	0
486	'FXYD2'	0.175	0
10538	'BATF'	0.17	0
3957	'LGALS2'	0.17	0
474381	'H2AB2'	0.17	0
55	'ACP3'	0.17	0
83740	'H2AB3'	0.17	0
100505841	'LOC100505841'	0.165	0
26212	'OR2B6'	0.165	0
5409	'PNMT'	0.16	0

101928268	'LOC101928268'	0.155	0
105376714	'LOC105376714'	0.15	0
54997	'TESC'	0.15	0
79852	'EPHX3'	0.15	0
102724652	'CRYAA2'	0.145	0
4993	'OR2C1'	0.145	0
55891	'LENEP'	0.145	0
6779	'STATH'	0.145	0
138255	'C9orf135'	0.14	0
6863	'TAC1'	0.14	0
112577461	'OOSP3'	0.135	0
100129836	'COL4A2-AS2'	0.13	0
149069	'DCDC2B'	0.125	0
339778	'FAM166C'	0.125	0
392133	'OR10AC1'	0.125	0
64581	'CLEC7A'	0.125	0
6588	'SLN'	0.125	0
11126	'CD160'	0.12	0
146556	'C16orf89'	0.12	0
4826	'NNAT'	0.12	0
64092	'SAMSN1'	0.12	0
150221	'RIMBP3C'	0.115	0
54979	'PLAAT2'	0.115	0
56287	'GKN1'	0.115	0
6013	'RLN1'	0.115	0
9071	'CLDN10'	0.115	0
100129543	'ZNF730'	0.11	0
105375106	'LOC105375106'	0.11	0
132243	'H1-8'	0.11	0
254187	'TSGA10IP'	0.11	0
282618	'IFNL1'	0.11	0
349334	'FOXD4L4'	0.11	0
5224	'PGAM2'	0.11	0
85291	'KRTAP4-2'	0.11	0
100505591	'LRRC3C'	0.105	0
100631383	'FAM47E-STBD1'	0.105	0
3050	'HBZ'	0.105	0
400891	'LRRC74B'	0.105	0
107987044	'LOC107987044'	0.1	0
145447	'ABHD12B'	0.1	0
340069	'FAM170A'	0.1	0
399888	'FAM180B'	0.1	0
440068	'CARD17'	0.1	0
55889	'GOLGA6B'	0.1	0
6357	'CCL13'	0.1	0
8399	'PLA2G10'	0.1	0
84066	'TEX35'	0.1	0
84460	'ZMAT1'	0.1	0
10158	'PDZK1IP1'	0.095	0
102723713	'LOC102723713'	0.095	0
29895	'MYLPF'	0.095	0
57829	'ZP4'	0.095	0
100526664	'LY75-CD302'	0.09	0
105372585	'LOC105372585'	0.09	0
129881	'CCDC173'	0.09	0
146325	'PRR35'	0.09	0

1504	'CTRB1'	0.09	0
2100	'ESR2'	0.09	0
23217	'ZFR2'	0.09	0
26330	'GAPDHS'	0.09	0
2780	'GNAT2'	0.09	0
285533	'RNF175'	0.09	0
326340	'ZAR1'	0.09	0
4050	'LTB'	0.09	0
440387	'CTRB2'	0.09	0
5617	'PRL'	0.09	0
608	'TNFRSF17'	0.09	0
644145	'EXOC1L'	0.09	0
80740	'LY6G6C'	0.09	0
84539	'MCHR2'	0.09	0
101928095	'LOC101928095'	0.085	0
107984745	'LOC107984745'	0.085	0
1259	'CNGA1'	0.085	0
151647	'TAFA4'	0.085	0
169166	'SNX31'	0.085	0
201516	'ZSCAN4'	0.085	0
51206	'GP6'	0.085	0
5733	'PTGER3'	0.085	0
7364	'UGT2B7'	0.085	0
8715	'NOL4'	0.085	0
100287284	'MANSC4'	0.08	0
105371095	'LOC105371095'	0.08	0
146279	'TEKT5'	0.08	0
148198	'ZNF98'	0.08	0
29944	'PNMA3'	0.08	0
387264	'KRTAP5-1'	0.08	0
390067	'OR52H1'	0.08	0
392309	'OR13J1'	0.08	0
399967	'PATE2'	0.08	0
90625	'ERVH48-1'	0.08	0
10361	'NPM2'	0.075	0
113220	'KIF12'	0.075	0
127550	'A3GALT2'	0.075	0
2793	'GNGT2'	0.075	0
283297	'OR10A4'	0.075	0
3570	'IL6R'	0.075	0
56163	'RNF17'	0.075	0
79785	'RERGL'	0.075	0
8778	'SIGLEC5'	0.075	0
105371045	'PERCC1'	0.07	0
107985734	'LOC107985734'	0.07	0
121214	'SDR9C7'	0.07	0
135946	'OR6B1'	0.07	0
162515	'SLC16A11'	0.07	0
3062	'HCRTR2'	0.07	0
343413	'FCRL6'	0.07	0
389692	'MAFA'	0.07	0
797	'CALCB'	0.07	0
92129	'RIPPLY1'	0.07	0
92483	'LDHAL6B'	0.07	0
100130302	'SUPT20HL1'	0.065	0
100419008	'PRR20G'	0.065	0

107985149	'LOC107985149'	0.065	0
107987175	'LOC107987175'	0.065	0
112267971	'LOC112267971'	0.065	0
135932	'TMEM139'	0.065	0
247	'ALOX15B'	0.065	0
2696	'GIPR'	0.065	0
340307	'CTAGE6'	0.065	0
388112	'NANOGP8'	0.065	0
390877	'LOC390877'	0.065	0
50831	'TAS2R3'	0.065	0
56158	'TEX12'	0.065	0
59272	'ACE2'	0.065	0
6539	'SLC6A12'	0.065	0
7139	'TNNT2'	0.065	0
79168	'LILRA6'	0.065	0
80831	'APOL5'	0.065	0
9402	'GRAP2'	0.065	0
100131303	'DPEP2NB'	0.06	0
100526761	'CCDC169-SOHLH2'	0.06	0
105370980	'LOC105370980'	0.06	0
151531	'UPP2'	0.06	0
151649	'PP2D1'	0.06	0
159963	'SLC5A12'	0.06	0
254439	'C11orf86'	0.06	0
27288	'RBMXL2'	0.06	0
29765	'TMOD4'	0.06	0
3822	'KLRC2'	0.06	0
49861	'CLDN20'	0.06	0
51151	'SLC45A2'	0.06	0
57348	'TTYH1'	0.06	0
643664	'SLC35G6'	0.06	0
100288072	'SDR42E2'	0.055	0
10149	'ADGRG2'	0.055	0
10875	'FGL2'	0.055	0
140	'ADORA3'	0.055	0
149095	'DCST1'	0.055	0
169693	'TMEM252'	0.055	0
2307	'FOXS1'	0.055	0
2833	'CXCR3'	0.055	0
285848	'PNPLA1'	0.055	0
2862	'MLNR'	0.055	0
55540	'IL17RB'	0.055	0
6441	'SFTPD'	0.055	0
84873	'ADGRG7'	0.055	0
102724265	'LOC102724265'	0.05	0
107984139	'LOC107984139'	0.05	0
107986531	'LOC107986531'	0.05	0
146849	'CCDC42'	0.05	0
221806	'VWDE'	0.05	0
284422	'SMIM24'	0.05	0
2914	'GRM4'	0.05	0
3270	'HRC'	0.05	0
345611	'IRGM'	0.05	0
3595	'IL12RB2'	0.05	0
375686	'SPATC1'	0.05	0
401613	'SERTM2'	0.05	0

4284	'MIP'	0.05	0
440050	'KRTAP5-7'	0.05	0
51802	'ASIC5'	0.05	0
5222	'PGA5'	0.05	0
5462	'POU5F1B'	0.05	0
643	'CXCR5'	0.05	0
7850	'IL1R2'	0.05	0
80133	'MROH9'	0.05	0
81025	'GJA9'	0.05	0
83650	'SLC35G5'	0.05	0
89790	'SIGLEC10'	0.05	0
91703	'ACY3'	0.05	0
100129654	'TCF24'	0.045	0
107987269	'LOC107987269'	0.045	0
374739	'TEPP'	0.045	0
3898	'LAD1'	0.045	0
440854	'CAPN14'	0.045	0
496	'ATP4B'	0.045	0
50700	'RDH8'	0.045	0
51179	'HAO2'	0.045	0
51412	'ACTL6B'	0.045	0
56244	'BTNL2'	0.045	0
5778	'PTPN7'	0.045	0
655	'BMP7'	0.045	0
728637	'MEIKIN'	0.045	0
7472	'WNT2'	0.045	0
766	'CA7'	0.045	0
84174	'SLA2'	0.045	0
9542	'NRG2'	0.045	0
100463285	'MTRNR2L4'	0.04	0
105379198	'LOC105379198'	0.04	0
112267940	'LOC112267940'	0.04	0
124842	'TMEM132E'	0.04	0
132625	'ZFP42'	0.04	0
169026	'SLC30A8'	0.04	0
25884	'CHRDL2'	0.04	0
2675	'GFRA2'	0.04	0
26762	'HAVCR1'	0.04	0
27329	'ANGPTL3'	0.04	0
279	'AMY2A'	0.04	0
284099	'C17orf78'	0.04	0
374569	'ASPG'	0.04	0
392188	'USP17L8'	0.04	0
400831	'C20orf202'	0.04	0
50614	'GALNT9'	0.04	0
51208	'CLDN18'	0.04	0
54905	'CYP2W1'	0.04	0
56670	'SUCNR1'	0.04	0
5673	'PSG5'	0.04	0
6097	'RORC'	0.04	0
6317	'SERPINB3'	0.04	0
643376	'BTBD18'	0.04	0
6474	'SHOX2'	0.04	0
83878	'USHBP1'	0.04	0
84103	'C4orf17'	0.04	0
84215	'ZNF541'	0.04	0

85366	'MYLK2'	0.04	0
9177	'HTR3B'	0.04	0
101927685	'HSFX4'	0.035	0
101928764	'LOC101928764'	0.035	0
101928917	'HSFX3'	0.035	0
101929372	'LOC101929372'	0.035	0
105375116	'LOC105375116'	0.035	0
116519	'APOA5'	0.035	0
1258	'CNGB1'	0.035	0
1439	'CSF2RB'	0.035	0
1496	'CTNNA2'	0.035	0
154661	'RUNDC3B'	0.035	0
1586	'CYP17A1'	0.035	0
171019	'ADAMTS19'	0.035	0
1804	'DPP6'	0.035	0
196472	'FAM71C'	0.035	0
2	'A2M'	0.035	0
219670	'ENKUR'	0.035	0
24141	'LAMP5'	0.035	0
2642	'GCGR'	0.035	0
27285	'TEKT2'	0.035	0
283375	'SLC39A5'	0.035	0
284254	'DYNAP'	0.035	0
3131	'HLF'	0.035	0
3375	'IAPP'	0.035	0
339967	'TMPRSS11A'	0.035	0
364	'AQP7'	0.035	0
3767	'KCNJ11'	0.035	0
377841	'ENTPD8'	0.035	0
4168	'MCF2'	0.035	0
55532	'SLC30A10'	0.035	0
56301	'SLC7A10'	0.035	0
570	'BAAT'	0.035	0
6783	'SULT1E1'	0.035	0
729747	'ZNF878'	0.035	0
79412	'KREMEN2'	0.035	0
80122	'MAP3K19'	0.035	0
81615	'TMEM163'	0.035	0
84630	'TTBK1'	0.035	0
9965	'FGF19'	0.035	0
999	'CDH1'	0.035	0
100132565	'GOLGA8F'	0.03	0
105369869	'LOC105369869'	0.03	0
105373944	'LOC105373944'	0.03	0
105379417	'LOC105379417'	0.03	0
106821730	'BUB1B-PAK6'	0.03	0
10736	'SIX2'	0.03	0
10753	'CAPN9'	0.03	0
11074	'TRIM31'	0.03	0
11081	'KERA'	0.03	0
1113	'CHGA'	0.03	0
1118	'CHIT1'	0.03	0
112267992	'LOC112267992'	0.03	0
117531	'TMC1'	0.03	0
131450	'CD200R1'	0.03	0
147920	'IGFL2'	0.03	0

1943	'EFNA2'	0.03	0
254272	'TBC1D28'	0.03	0
26280	'IL1RAPL2'	0.03	0
2676	'GFRA3'	0.03	0
2912	'GRM2'	0.03	0
319089	'TTC6'	0.03	0
338442	'HCAR2'	0.03	0
340895	'MALRD1'	0.03	0
346606	'MOGAT3'	0.03	0
3483	'IGFALS'	0.03	0
3739	'KCNA4'	0.03	0
399948	'COLCA1'	0.03	0
408263	'FNDC9'	0.03	0
445582	'POTEE'	0.03	0
4625	'MYH7'	0.03	0
51411	'BIN2'	0.03	0
53841	'CDHR5'	0.03	0
54112	'GPR88'	0.03	0
54836	'BSPRY'	0.03	0
56165	'TDRD1'	0.03	0
57642	'COL20A1'	0.03	0
6000	'RGS7'	0.03	0
64407	'RGS18'	0.03	0
645121	'CCNI2'	0.03	0
6519	'SLC3A1'	0.03	0
6527	'SLC5A4'	0.03	0
6554	'SLC10A1'	0.03	0
695	'BTK'	0.03	0
729020	'RPEL1'	0.03	0
732	'C8B'	0.03	0
79825	'EFCC1'	0.03	0
79849	'PDZD3'	0.03	0
81626	'SHCBP1L'	0.03	0
8843	'HCAR3'	0.03	0
90249	'UNC5A'	0.03	0
9283	'GPR37L1'	0.03	0
9332	'CD163'	0.03	0
105372319	'LOC105372319'	0.025	0
107985657	'LOC107985657'	0.025	0
107987289	'LOC107987289'	0.025	0
168090	'C6orf118'	0.025	0
169355	'IDO2'	0.025	0
2078	'ERG'	0.025	0
2359	'FPR3'	0.025	0
242	'ALOX12B'	0.025	0
2662	'GDF10'	0.025	0
27098	'CLUL1'	0.025	0
284541	'CYP4A22'	0.025	0
2913	'GRM3'	0.025	0
3248	'HPGD'	0.025	0
339398	'LINGO4'	0.025	0
3683	'ITGAL'	0.025	0
3694	'ITGB6'	0.025	0
3754	'KCNF1'	0.025	0
392843	'IQCA1L'	0.025	0
4129	'MAOB'	0.025	0

4340	'MOG'	0.025	0
54715	'RBFOX1'	0.025	0
55211	'DPPA4'	0.025	0
56134	'PCDHAC2'	0.025	0
56673	'C11orf16'	0.025	0
57101	'ANO2'	0.025	0
63973	'NEUROG2'	0.025	0
643677	'CCDC168'	0.025	0
6693	'SPN'	0.025	0
793	'CALB1'	0.025	0
80736	'SLC44A4'	0.025	0
80834	'TAS1R2'	0.025	0
84808	'PERM1'	0.025	0
94233	'OPN4'	0.025	0
1001	'CDH3'	0.02	0
1006	'CDH8'	0.02	0
105370691	'LOC105370691'	0.02	0
10878	'CFHR3'	0.02	0
10990	'LILRB5'	0.02	0
112267910	'LOC112267910'	0.02	0
1136	'CHRNA3'	0.02	0
114824	'PNMA5'	0.02	0
1233	'CCR4'	0.02	0
125931	'CEACAM20'	0.02	0
144423	'GLT1D1'	0.02	0
147138	'TMC8'	0.02	0
161829	'EXD1'	0.02	0
163479	'FNDC7'	0.02	0
221472	'FGD2'	0.02	0
222546	'RFX6'	0.02	0
22953	'P2RX2'	0.02	0
23120	'ATP10B'	0.02	0
2516	'NR5A1'	0.02	0
26683	'OR4F3'	0.02	0
27023	'FOXB1'	0.02	0
27071	'DAPP1'	0.02	0
27134	'TJP3'	0.02	0
3356	'HTR2A'	0.02	0
340267	'COL28A1'	0.02	0
340419	'RSPO2'	0.02	0
340595	'RTL4'	0.02	0
342667	'STAC2'	0.02	0
3777	'KCNK3'	0.02	0
441308	'OR4F21'	0.02	0
494551	'WEE2'	0.02	0
5350	'PLN'	0.02	0
5535	'PPP3R2'	0.02	0
55503	'TRPV6'	0.02	0
56128	'PCDHB8'	0.02	0
56667	'MUC13'	0.02	0
56853	'CELF4'	0.02	0
57156	'TMEM63C'	0.02	0
575	'ADGRB1'	0.02	0
6010	'RHO'	0.02	0
643394	'SPINK9'	0.02	0
644943	'RASSF10'	0.02	0

6564	'SLC15A1'	0.02	0
696	'BTN1A1'	0.02	0
729759	'OR4F29'	0.02	0
79958	'DENND1C'	0.02	0
8074	'FGF23'	0.02	0
84229	'DRC7'	0.02	0
84684	'INSM2'	0.02	0
8521	'GCM1'	0.02	0
85508	'SCRT2'	0.02	0
8735	'MYH13'	0.02	0
90485	'ZNF835'	0.02	0
944	'TNFSF8'	0.02	0
101929805	'LOC101929805'	0.015	0
11027	'LILRA2'	0.015	0
115350	'FCRL1'	0.015	0
1261	'CNGA3'	0.015	0
139105	'BEND2'	0.015	0
139221	'PWWP3B'	0.015	0
146395	'GSG1L'	0.015	0
1812	'DRD1'	0.015	0
23533	'PIK3R5'	0.015	0
257629	'ANKS4B'	0.015	0
27091	'CACNG5'	0.015	0
27092	'CACNG4'	0.015	0
27127	'SMC1B'	0.015	0
27201	'GPR78'	0.015	0
283284	'IGSF22'	0.015	0
283576	'ZDHHC22'	0.015	0
2844	'GPR21'	0.015	0
440077	'ZNF705A'	0.015	0
441151	'TMEM151B'	0.015	0
4585	'MUC4'	0.015	0
5076	'PAX2'	0.015	0
55911	'APOBR'	0.015	0
57582	'KCNT1'	0.015	0
57795	'BRINP2'	0.015	0
6011	'GRK1'	0.015	0
6330	'SCN4B'	0.015	0
63923	'TNN'	0.015	0
79838	'TMC5'	0.015	0
79865	'TREML2'	0.015	0
80059	'LRRTM4'	0.015	0
91683	'SYT12'	0.015	0
102724631	'POTEB3'	0.01	0
105370849	'LOC105370849'	0.01	0
107985876	'LOC107985876'	0.01	0
1080	'CFTR'	0.01	0
158866	'ZDHHC15'	0.01	0
205147	'AMER3'	0.01	0
2104	'ESRRG'	0.01	0
23316	'CUX2'	0.01	0
26034	'IPCEF1'	0.01	0
27198	'HCAR1'	0.01	0
284805	'C20orf203'	0.01	0
2911	'GRM1'	0.01	0
338	'APOB'	0.01	0

339761	'CYP27C1'	0.01	0
342850	'ANKRD62'	0.01	0
3745	'KCNB1'	0.01	0
375323	'LHFPL4'	0.01	0
401720	'FIGNL2'	0.01	0
4036	'LRP2'	0.01	0
477	'ATP1A2'	0.01	0
50506	'DUOX2'	0.01	0
54798	'DCHS2'	0.01	0
6332	'SCN7A'	0.01	0
730	'C7'	0.01	0
776	'CACNA1D'	0.01	0
8128	'ST8SIA2'	0.01	0
8972	'MGAM'	0.01	0
92211	'CDHR1'	0.01	0
9615	'GDA'	0.01	0
10178	'TENM1'	0.005	0
2153	'F5'	0.005	0
2258	'FGF13'	0.005	0
25769	'SLC24A2'	0.005	0
26154	'ABCA12'	0.005	0
284312	'ZSCAN1'	0.005	0
285513	'GPRIN3'	0.005	0
340990	'OTOG'	0.005	0
4142	'MAS1'	0.005	0
442319	'ZNF727'	0.005	0
4583	'MUC2'	0.005	0
4584	'MUC3A'	0.005	0
5178	'PEG3'	0.005	0
727897	'MUC5B'	0.005	0
7757	'ZNF208'	0.005	0
84059	'ADGRV1'	0.005	0

Supplementary Table 5. DEGs between CD317+ vs CD317- MSCs

Table S5.3. Expressed genes only in CD317- MSCs

Gene ID	Gene Symbol	Average FPKM CD317-	Average FPKM CD317+
5032	'P2RY11'	2.21	0
255411	'TEX49'	1.59	0
10344	'CCL26'	1.225	0
641517	'DEFB109B'	0.975	0
110354863	'ZNF660-ZNF197'	0.555	0
644186	'SYCE3'	0.435	0
92304	'SCGB3A1'	0.41	0
101927655	'ZASP'	0.4	0
29802	'VPREB3'	0.38	0
8355	'H3C8'	0.25	0
8358	'H3C2'	0.23	0
27290	'SPINK4'	0.215	0
8294	'H4C9'	0.215	0
5121	'PCP4'	0.21	0
8364	'H4C3'	0.205	0
124404	'SEPTIN12'	0.195	0
6289	'SAA2'	0.18	0
100129480	'MKRN2OS'	0.175	0
50840	'TAS2R14'	0.175	0
100529251	'CKLF-CMTM1'	0.17	0
3007	'H1-3'	0.165	0
119467	'CLRN3'	0.16	0
360205	'PRAC2'	0.16	0
1448	'CSN3'	0.155	0
342538	'NACA2'	0.155	0
8340	'H2BC13'	0.155	0
100506540	'SPTY2D1OS'	0.15	0
123346	'HIGD2B'	0.15	0
4635	'MYL4'	0.145	0
3816	'KLK1'	0.14	0
8344	'H2BC6'	0.14	0
100534592	'URGCP-MRPS24'	0.135	0
7136	'TNNI2'	0.135	0
8346	'H2BC10'	0.135	0
285588	'EFCAB9'	0.13	0
84417	'ECRG4'	0.13	0
8993	'PGLYRP1'	0.13	0
392138	'OR2A25'	0.125	0
50632	'CALY'	0.115	0
8350	'H3C1'	0.115	0
8356	'H3C12'	0.115	0
132332	'TMEM155'	0.11	0
1469	'CST1'	0.11	0
100130742	'LRRC69'	0.105	0
119774	'OR52K2'	0.105	0
165545	'DQX1'	0.105	0
284417	'TMEM150B'	0.105	0
284427	'SLC25A41'	0.105	0
341947	'COX8C'	0.105	0
440585	'FAM183A'	0.105	0
5630	'PRPH'	0.105	0

158798	'AKAP14'	0.1	0
5319	'PLA2G1B'	0.1	0
27156	'RSPH14'	0.095	0
388799	'FAM209B'	0.095	0
55908	'ANGPTL8'	0.095	0
100529215	'ZNF559-ZNF177'	0.09	0
102724197	'LOC102724197'	0.09	0
352999	'C6orf58'	0.09	0
374403	'TBC1D10C'	0.09	0
399	'RHOH'	0.09	0
434	'ASIP'	0.09	0
474382	'H2AB1'	0.09	0
10911	'UTS2'	0.085	0
259	'AMBP'	0.085	0
285555	'STPG2'	0.085	0
552891	'DNAJC25-GNG10'	0.085	0
56891	'LGALS14'	0.085	0
100996598	'LOC100996598'	0.08	0
1776	'DNASE1L3'	0.08	0
206412	'C6orf163'	0.08	0
353164	'TAS2R42'	0.08	0
6690	'SPINK1'	0.08	0
729201	'SPACA5B'	0.08	0
9535	'GMFG'	0.08	0
100132463	'CLDN24'	0.075	0
101928677	'ETDA'	0.075	0
23563	'CHST5'	0.075	0
647219	'ASCL5'	0.075	0
94115	'CGB8'	0.075	0
100129128	'KHDC1L'	0.07	0
101927572	'LOC101927572'	0.07	0
375298	'CERKL'	0.07	0
390191	'OR5B12'	0.07	0
57111	'RAB25'	0.07	0
6563	'SLC14A1'	0.07	0
6954	'TCP11'	0.07	0
105372412	'LOC105372412'	0.065	0
131831	'ERICH6'	0.065	0
399949	'C11orf88'	0.065	0
6037	'RNASE3'	0.065	0
644974	'ALG1L2'	0.065	0
100874261	'CCDC200'	0.06	0
101929400	'LOC101929400'	0.06	0
102724862	'TBC1D3I'	0.06	0
10744	'PTTG2'	0.06	0
107984640	'LBHD2'	0.06	0
129852	'C2orf73'	0.06	0
146183	'OTOA'	0.06	0
162466	'PHOSPHO1'	0.06	0
3010	'H1-6'	0.06	0
431705	'ASTL'	0.06	0
57016	'AKR1B10'	0.06	0
6123	'RPL3L'	0.06	0
7123	'CLEC3B'	0.06	0
8435	'SOAT2'	0.06	0
92359	'CRB3'	0.06	0

102724231	'C3orf86'	0.055	0
146310	'RNF151'	0.055	0
170589	'GPHA2'	0.055	0
340205	'TREML1'	0.055	0
344805	'TMPRSS7'	0.055	0
388585	'HES5'	0.055	0
50604	'IL20'	0.055	0
51207	'DUSP13'	0.055	0
6349	'CCL3L1'	0.055	0
107984859	'LOC107984859'	0.05	0
124912	'SPACA3'	0.05	0
267020	'ATP5MGL'	0.05	0
282808	'RAB40AL'	0.05	0
388325	'SCIMP'	0.05	0
3934	'LCN2'	0.05	0
401335	'C7orf65'	0.05	0
4878	'NPPA'	0.05	0
54831	'BEST2'	0.05	0
58524	'DMRT3'	0.05	0
83597	'RTP3'	0.05	0
100653133	'LOC100653133'	0.045	0
101930434	'LOC101930434'	0.045	0
105377310	'LOC105377310'	0.045	0
120224	'TMEM45B'	0.045	0
1417	'CRYBB3'	0.045	0
146225	'CMTM2'	0.045	0
146845	'CFAP52'	0.045	0
2167	'FABP4'	0.045	0
285231	'FBXW12'	0.045	0
388649	'C1orf146'	0.045	0
445372	'TRIM6-TRIM34'	0.045	0
55286	'C4orf19'	0.045	0
84675	'TRIM55'	0.045	0
84824	'FCRLA'	0.045	0
8838	'CCN6'	0.045	0
107985555	'LOC107985555'	0.04	0
10858	'CYP46A1'	0.04	0
116173	'CMTM5'	0.04	0
171169	'SPACA4'	0.04	0
259296	'TAS2R50'	0.04	0
27287	'VENTX'	0.04	0
284110	'GSDMA'	0.04	0
285525	'YIPF7'	0.04	0
29949	'IL19'	0.04	0
390093	'OR10A6'	0.04	0
5030	'P2RY4'	0.04	0
54578	'UGT1A6'	0.04	0
54586	'EQTN'	0.04	0
56132	'PCDHB3'	0.04	0
728858	'C12orf71'	0.04	0
7546	'ZIC2'	0.04	0
81392	'OR2AE1'	0.04	0
9499	'MYOT'	0.04	0
102723796	'TLE7'	0.035	0
10345	'TRDN'	0.035	0
1149	'CIDEA'	0.035	0

131540	'ZDHHC19'	0.035	0
144448	'TSPAN19'	0.035	0
255101	'CFAP65'	0.035	0
284359	'IZUMO1'	0.035	0
284521	'OR2L13'	0.035	0
3228	'HOXC12'	0.035	0
359787	'DPPA3'	0.035	0
4151	'MB'	0.035	0
440021	'KRTAP5-2'	0.035	0
58511	'DNASE2B'	0.035	0
7111	'TMOD1'	0.035	0
7201	'TRHR'	0.035	0
93492	'TPTE2'	0.035	0
100130933	'SMIM6'	0.03	0
107986910	'LOC107986910'	0.03	0
124783	'SPATA32'	0.03	0
159296	'NKX2-3'	0.03	0
164668	'APOBEC3H'	0.03	0
220047	'CCDC83'	0.03	0
23584	'VSIG2'	0.03	0
254268	'AKNAD1'	0.03	0
285489	'DOK7'	0.03	0
2893	'GRIA4'	0.03	0
3512	'JCHAIN'	0.03	0
3758	'KCNJ1'	0.03	0
3780	'KCNN1'	0.03	0
401399	'PRRT4'	0.03	0
5454	'POU3F2'	0.03	0
56961	'SHD'	0.03	0
57159	'TRIM54'	0.03	0
57830	'KRTAP5-8'	0.03	0
64098	'PARVG'	0.03	0
6579	'SLCO1A2'	0.03	0
6725	'SRMS'	0.03	0
7512	'XPNPEP2'	0.03	0
81551	'STMN4'	0.03	0
84692	'CCDC54'	0.03	0
84970	'C1orf94'	0.03	0
9075	'CLDN2'	0.03	0
974	'CD79B'	0.03	0
100505502	'LOC100505502'	0.025	0
10242	'KCNMB2'	0.025	0
132228	'LSMEM2'	0.025	0
133491	'C5orf47'	0.025	0
140690	'CTCFL'	0.025	0
140902	'R3HDML'	0.025	0
147407	'SLC25A52'	0.025	0
165100	'TEX44'	0.025	0
202151	'RANBP3L'	0.025	0
27006	'FGF22'	0.025	0
27039	'PKD2L2'	0.025	0
2841	'GPR18'	0.025	0
341880	'SLC35F4'	0.025	0
375307	'CATIP'	0.025	0
400823	'FAM177B'	0.025	0
51361	'HOOK1'	0.025	0

51778	'MYOZ2'	0.025	0
5972	'REN'	0.025	0
643136	'ZC3H11B'	0.025	0
646892	'SH2D7'	0.025	0
653781	'POTEJ'	0.025	0
7053	'TGM3'	0.025	0
727857	'BHLHA9'	0.025	0
80168	'MOGAT2'	0.025	0
80342	'TRAF3IP3'	0.025	0
92558	'BICDL1'	0.025	0
9478	'CABP1'	0.025	0
100507341	'SMIM18'	0.02	0
1044	'CDX1'	0.02	0
105370733	'LOC105370733'	0.02	0
11005	'SPINK5'	0.02	0
114905	'C1QTNF7'	0.02	0
118663	'BTBD16'	0.02	0
135250	'RAET1E'	0.02	0
158830	'CXorf65'	0.02	0
160287	'LDHAL6A'	0.02	0
196374	'KRT78'	0.02	0
199699	'DAND5'	0.02	0
2301	'FOXE3'	0.02	0
23440	'OTP'	0.02	0
23746	'AIPL1'	0.02	0
254158	'CXorf58'	0.02	0
256297	'PTF1A'	0.02	0
257062	'CATSPERD'	0.02	0
257240	'KLHL34'	0.02	0
2857	'GPR34'	0.02	0
3003	'GZMK'	0.02	0
345274	'SLC10A6'	0.02	0
345930	'ECT2L'	0.02	0
363	'AQP6'	0.02	0
374877	'TEX45'	0.02	0
375607	'NAT16'	0.02	0
3881	'KRT31'	0.02	0
388135	'INSYN1'	0.02	0
442444	'FAM47C'	0.02	0
4693	'NDP'	0.02	0
5013	'OTX1'	0.02	0
548596	'CKMT1A'	0.02	0
57096	'RPGRIP1'	0.02	0
64174	'DPEP2'	0.02	0
64211	'LHX5'	0.02	0
6456	'SH3GL2'	0.02	0
646799	'ZAR1L'	0.02	0
64926	'RASAL3'	0.02	0
6975	'TECTB'	0.02	0
7941	'PLA2G7'	0.02	0
8631	'SKAP1'	0.02	0
969	'CD69'	0.02	0
11023	'VAX1'	0.015	0
11309	'SLCO2B1'	0.015	0
151112	'ZSWIM2'	0.015	0
153743	'PPP1R2B'	0.015	0

155051	'CRYGN'	0.015	0
167127	'UGT3A2'	0.015	0
170261	'ZCCHC12'	0.015	0
170591	'S100Z'	0.015	0
1735	'DIO3'	0.015	0
219681	'ARMC3'	0.015	0
219972	'MPEG1'	0.015	0
23504	'RIMBP2'	0.015	0
253012	'HEPACAM2'	0.015	0
284904	'SEC14L4'	0.015	0
340273	'ABCB5'	0.015	0
3549	'IHH'	0.015	0
3645	'INSRR'	0.015	0
3670	'ISL1'	0.015	0
389058	'SP5'	0.015	0
389152	'PRR23C'	0.015	0
389643	'NUGGC'	0.015	0
392490	'FLJ44635'	0.015	0
3973	'LHCGR'	0.015	0
4145	'MATK'	0.015	0
440603	'BCL2L15'	0.015	0
442721	'LMOD2'	0.015	0
5105	'PCK1'	0.015	0
51305	'KCNK9'	0.015	0
51617	'NSG2'	0.015	0
5173	'PDYN'	0.015	0
55259	'CFAP94'	0.015	0
56124	'PCDHB12'	0.015	0
5745	'PTH1R'	0.015	0
635	'BHMT'	0.015	0
646951	'MINDY4B'	0.015	0
64919	'BCL11B'	0.015	0
6532	'SLC6A4'	0.015	0
6549	'SLC9A2'	0.015	0
6572	'SLC18A3'	0.015	0
7287	'TULP1'	0.015	0
7363	'UGT2B4'	0.015	0
7471	'WNT1'	0.015	0
7704	'ZBTB16'	0.015	0
79413	'ZBED2'	0.015	0
796	'CALCA'	0.015	0
79645	'EFCAB1'	0.015	0
81492	'RSPH6A'	0.015	0
8328	'GFI1B'	0.015	0
84189	'SLITRK6'	0.015	0
85439	'STON2'	0.015	0
90665	'TBL1Y'	0.015	0
91156	'IGFN1'	0.015	0
9153	'SLC28A2'	0.015	0
9248	'GPR50'	0.015	0
925	'CD8A'	0.015	0
1015	'CDH17'	0.01	0
10451	'VAV3'	0.01	0
105371242	'PPIAL4H'	0.01	0
105373985	'LOC105373985'	0.01	0
105377622	'LOC105377622'	0.01	0

105378952	'KLF18'	0.01	0
10595	'ERN2'	0.01	0
10716	'TBR1'	0.01	0
107987211	'LOC107987211'	0.01	0
107987285	'LOC107987285'	0.01	0
11086	'ADAM29'	0.01	0
11189	'CELF3'	0.01	0
116	'ADCYAP1'	0.01	0
121643	'FOXN4'	0.01	0
124857	'WFIKKN2'	0.01	0
125115	'KRT40'	0.01	0
126669	'SHE'	0.01	0
127343	'DMBX1'	0.01	0
129446	'XIRP2'	0.01	0
130	'ADH6'	0.01	0
138065	'RNF183'	0.01	0
140679	'SLC32A1'	0.01	0
146212	'KCTD19'	0.01	0
148870	'CCDC27'	0.01	0
1501	'CTNND2'	0.01	0
152404	'IGSF11'	0.01	0
153020	'RASGEF1B'	0.01	0
170302	'ARX'	0.01	0
189	'AGXT'	0.01	0
1995	'ELAVL3'	0.01	0
2046	'EPHA8'	0.01	0
2165	'F13B'	0.01	0
220965	'FAM13C'	0.01	0
23263	'MCF2L'	0.01	0
23415	'KCNH4'	0.01	0
23418	'CRB1'	0.01	0
246176	'GAS2L2'	0.01	0
26468	'LHX6'	0.01	0
2660	'MSTN'	0.01	0
2693	'GHSR'	0.01	0
27087	'B3GAT1'	0.01	0
283847	'TERB1'	0.01	0
2845	'GPR22'	0.01	0
284525	'SLC9C2'	0.01	0
3101	'HK3'	0.01	0
340562	'SATL1'	0.01	0
343702	'XKR7'	0.01	0
343990	'CRACDL'	0.01	0
3579	'CXCR2'	0.01	0
389257	'LRRC14B'	0.01	0
390748	'PABPN1L'	0.01	0
401265	'KLHL31'	0.01	0
401387	'LRRD1'	0.01	0
440822	'PIWIL3'	0.01	0
4626	'MYH8'	0.01	0
474344	'GIMAP6'	0.01	0
50834	'TAS2R1'	0.01	0
5354	'PLP1'	0.01	0
5470	'PPEF2'	0.01	0
55040	'EPN3'	0.01	0
5579	'PRKCB'	0.01	0

59084	'ENPP5'	0.01	0
6101	'RP1'	0.01	0
64072	'CDH23'	0.01	0
642636	'RAD21L1'	0.01	0
6439	'SFTPB'	0.01	0
65055	'REEP1'	0.01	0
6517	'SLC2A4'	0.01	0
6534	'SLC6A7'	0.01	0
653643	'GOLGA6D'	0.01	0
66037	'BOLL'	0.01	0
7062	'TCHH'	0.01	0
7135	'TNNI1'	0.01	0
7166	'TPH1'	0.01	0
729025	'SLC15A5'	0.01	0
729475	'RAD51AP2'	0.01	0
729956	'SHISA7'	0.01	0
7429	'VIL1'	0.01	0
7712	'ZNF157'	0.01	0
79190	'IRX6'	0.01	0
79400	'NOX5'	0.01	0
79755	'ZNF750'	0.01	0
79843	'FAM124B'	0.01	0
827	'CAPN6'	0.01	0
8288	'EPX'	0.01	0
84063	'KIRREL2'	0.01	0
84109	'QRFPR'	0.01	0
84528	'RHOXF2'	0.01	0
89792	'GAL3ST3'	0.01	0
91662	'NLRP12'	0.01	0
9340	'GLP2R'	0.01	0
9427	'ECEL1'	0.01	0
100506164	'HSFX1'	0.005	0
105374103	'LOC105374103'	0.005	0
105378947	'LOC105378947'	0.005	0
107985692	'LOC107985692'	0.005	0
107986982	'LOC107986982'	0.005	0
1268	'CNR1'	0.005	0
140803	'TRPM6'	0.005	0
1536	'CYBB'	0.005	0
2125	'EVPL'	0.005	0
219287	'AMER2'	0.005	0
23105	'FSTL4'	0.005	0
2538	'G6PC'	0.005	0
254773	'LYG2'	0.005	0
259308	'FAM205A'	0.005	0
27143	'PALD1'	0.005	0
283521	'TMEM272'	0.005	0
285596	'FAM153A'	0.005	0
3662	'IRF4'	0.005	0
3684	'ITGAM'	0.005	0
392862	'GRID2IP'	0.005	0
4064	'CD180'	0.005	0
4607	'MYBPC3'	0.005	0
4648	'MYO7B'	0.005	0
491	'ATP2B2'	0.005	0
55966	'AJAP1'	0.005	0

57105	'CYSLTR2'	0.005	0
57282	'SLC4A10'	0.005	0
57530	'CGN'	0.005	0
5788	'PTPRC'	0.005	0
5949	'RBP3'	0.005	0
619279	'ZNF704'	0.005	0
64106	'NPFFR1'	0.005	0
641455	'POTEM'	0.005	0
64241	'ABCG8'	0.005	0
643236	'TMEM72'	0.005	0
651	'BMP3'	0.005	0
6860	'SYT4'	0.005	0
7100	'TLR5'	0.005	0
7224	'TRPC5'	0.005	0
79054	'TRPM8'	0.005	0
79923	'NANOG'	0.005	0
83539	'CHST9'	0.005	0
84443	'FRMPD3'	0.005	0
84502	'JPH4'	0.005	0
85300	'ATCAY'	0.005	0
9381	'OTOF'	0.005	0
9717	'SEC14L5'	0.005	0
338321	'NLRP9'	0.0001	0
3782	'KCNN3'	0.0001	0
56139	'PCDHA10'	0.0001	0
6323	'SCN1A'	0.0001	0
